# Addressing Global Ruminant Agricultural Challenges Through Understanding the Rumen Microbiome: Past, Present, and Future

**DOI:** 10.3389/fmicb.2018.02161

**Published:** 2018-09-25

**Authors:** Sharon A. Huws, Christopher J. Creevey, Linda B. Oyama, Itzhak Mizrahi, Stuart E. Denman, Milka Popova, Rafael Muñoz-Tamayo, Evelyne Forano, Sinead M. Waters, Matthias Hess, Ilma Tapio, Hauke Smidt, Sophie J. Krizsan, David R. Yáñez-Ruiz, Alejandro Belanche, Leluo Guan, Robert J. Gruninger, Tim A. McAllister, C. Jamie Newbold, Rainer Roehe, Richard J. Dewhurst, Tim J. Snelling, Mick Watson, Garret Suen, Elizabeth H. Hart, Alison H. Kingston-Smith, Nigel D. Scollan, Rodolpho M. do Prado, Eduardo J. Pilau, Hilario C. Mantovani, Graeme T. Attwood, Joan E. Edwards, Neil R. McEwan, Steven Morrisson, Olga L. Mayorga, Christopher Elliott, Diego P. Morgavi

**Affiliations:** ^1^Institute for Global Food Security, Queen's University of Belfast, Belfast, United Kingdom; ^2^Department of Life Sciences and the National Institute for Biotechnology in the Negev, Ben Gurion University of the Negev, Beer Sheva, Israel; ^3^Commonwealth Scientific and Industrial Research Organisation Agriculture and Food, Queensland Bioscience Precinct, St Lucia, QLD, Australia; ^4^Institute National de la Recherche Agronomique, UMR1213 Herbivores, Clermont Université, VetAgro Sup, UMR Herbivores, Clermont-Ferrand, France; ^5^UMR Modélisation Systémique Appliquée aux Ruminants, INRA, AgroParisTech, Université Paris-Saclay, Paris, France; ^6^UMR 454 MEDIS, INRA, Université Clermont Auvergne, Clermont-Ferrand, France; ^7^Animal and Bioscience Research Department, Animal and Grassland Research and Innovation Centre, Grange, Ireland; ^8^College of Agricultural and Environmental Sciences, University of California, Davis, Davis, CA, United States; ^9^Natural Resources Institute Finland, Jokioinen, Finland; ^10^Department of Agrotechnology and Food Sciences, Wageningen, Netherlands; ^11^Department of Agricultural Research for Northern Sweden, Swedish University of Agricultural Sciences, Umeå, Sweden; ^12^Estacion Experimental del Zaidin, Consejo Superior de Investigaciones Cientificas, Granada, Spain; ^13^Department of Agricultural, Food and Nutritional Science, University of Alberta, Edmonton, AB, Canada; ^14^Lethbridge Research Centre, Agriculture and Agri-Food Canada, Lethbridge, AB, Canada; ^15^Scotland's Rural College, Edinburgh, United Kingdom; ^16^The Rowett Institute, University of Aberdeen, Aberdeen, United Kingdom; ^17^The Roslin Institute and the Royal (Dick) School of Veterinary Studies (R(D)SVS), University of Edinburgh, Edinburgh, United Kingdom; ^18^Department of Bacteriology, University of Wisconsin-Madison, Madison, WI, United States; ^19^Institute of Biological, Environmental and Rural Sciences, Aberystwyth University, Aberystwyth, United Kingdom; ^20^Laboratório de Biomoléculas e Espectrometria de Massas-Labiomass, Departamento de Química, Universidade Estadual de Maringá, Maringá, Brazil; ^21^Department of Microbiology, Universidade Federal de Viçosa, Viçosa, Brazil; ^22^AgResearch Limited, Grasslands Research Centre, Palmerston North, New Zealand; ^23^Laboratory of Microbiology, Wageningen University & Research, Wageningen, Netherlands; ^24^School of Pharmacy and Life Sciences, Robert Gordon University, Aberdeen, United Kingdom; ^25^Sustainable Livestock, Agri-Food and Bio-Sciences Institute, Hillsborough, United Kingdom; ^26^Colombian Agricultural Research Corporation, Mosquera, Colombia

**Keywords:** rumen, microbiome, host, diet, production, methane, omics

## Abstract

The rumen is a complex ecosystem composed of anaerobic bacteria, protozoa, fungi, methanogenic archaea and phages. These microbes interact closely to breakdown plant material that cannot be digested by humans, whilst providing metabolic energy to the host and, in the case of archaea, producing methane. Consequently, ruminants produce meat and milk, which are rich in high-quality protein, vitamins and minerals, and therefore contribute to food security. As the world population is predicted to reach approximately 9.7 billion by 2050, an increase in ruminant production to satisfy global protein demand is necessary, despite limited land availability, and whilst ensuring environmental impact is minimized. Although challenging, these goals can be met, but depend on our understanding of the rumen microbiome. Attempts to manipulate the rumen microbiome to benefit global agricultural challenges have been ongoing for decades with limited success, mostly due to the lack of a detailed understanding of this microbiome and our limited ability to culture most of these microbes outside the rumen. The potential to manipulate the rumen microbiome and meet global livestock challenges through animal breeding and introduction of dietary interventions during early life have recently emerged as promising new technologies. Our inability to phenotype ruminants in a high-throughput manner has also hampered progress, although the recent increase in “omic” data may allow further development of mathematical models and rumen microbial gene biomarkers as proxies. Advances in computational tools, high-throughput sequencing technologies and cultivation-independent “omics” approaches continue to revolutionize our understanding of the rumen microbiome. This will ultimately provide the knowledge framework needed to solve current and future ruminant livestock challenges.

## Global agricultural challenges

There are currently 7.5 billion humans on the planet, and the world Hunger Map estimates that 795 million people (over 10%) do not have access to sufficient food (WFP, 2015). Whilst some models predict the world population to peak at 9.7 billion in 2050, others estimate a population of 11.2 billion in 2100 (United Nations, 2015). To meet an increasing demand for food, the Food and Agriculture Organization of the United Nations (FAO) predicts that total agricultural production (including crops and animals) will need to be 60% higher than in 2005. With animal protein demand rising at a proportionally faster rate, estimates suggest that global meat and milk production will have to increase by 76 and 63%, respectively (Alexandratos and Bruinsma, 2012).

This extensive population growth, coupled with an increased consumption of ruminant products by developing countries, will add to the strain on the availability of safe and nutritious ruminant products. Due to land constraints, the number of pastured ruminants cannot increase and therefore efforts should be directed toward increasing production efficiency. Indeed, efficient utilization of feed by the rumen microbiome results in enhanced nutrient availability to the host, and thus improved production efficiency is central to ensuring food security. Feed for ruminants typically accounts for 60–70% of total expenditure in beef production (Karisa et al., 2014; Fouhse et al., 2017), whilst requiring substantial land mass for plant growth. Residual feed intake (RFI), which is the difference between the predicted (based on energy demands) and actual intake, has been proposed as a more meaningful measure for calculating feed efficiency (Berry and Crowley, 2012; Shabat et al., 2016). RFI values of 1.45 (high RFI) and −1.64 kg/day (low RFI) have been noted for crossbred steers (with 0 being the expected and values <0 inferring that the animal has greater feed efficiency than expected), resulting in high RFI animals requiring approximately 1,000 kg more feed/annum than low RFI animals to achieve the same production parameters (Fouhse et al., 2017). Therefore, understanding the underlying mechanisms for RFI, particularly with respect to the involvement of the rumen microbiome, could aid efficiency and sustainability of ruminant production (Mizrahi, 2011).

Ruminant livestock production has been estimated to be responsible for approximately 14% of anthropogenic methane, a potent greenhouse gas (GHG), released annually into the atmosphere due to the activity of rumen methanogens (Gerber et al., 2013). The released methane, produced by rumen methanogens, is a major problem for the environment, but also a great concern to livestock production as around 2–8% of the dietary energy can be lost to methane (CH_4_) production (IPCC, 2006); values as high as 12% have been reported for low quality feeds (Johnson and Johnson, 1995). Nonetheless, reductions in methane emissions do not always result in a redirection of energy, leading to enhanced animal production. For example, 3-nitrooxypropanol (3-NOP) has been shown to reduce methane emissions by up to 30% (Hristov et al., 2015; Jayanegara et al., 2018). However, a meta-analysis of all available animal data following supplementation with 3-NOP only shows modest increases in animal production, possibly due to decreased volatile fatty acid (VFA) produced from breakdown of cellulose and increased H_2_ production; both processes requiring energy input (Jayanegara et al., 2018).

The rumen microbiome is also pivotal to nitrogen (N) use efficiency due to its role in proteolysis and catabolism of amino acids, resulting in microbial N, which contributes 60–90% of protein absorbed at the duodenum (Wallace et al., 1997). Ruminant N use efficiency also needs to be improved to optimize production and reduce the environmental footprint of the industry as ruminants excrete approximately 70% of ingested N (Macrae and Ulyatt, 1974; Dewhurst et al., 1996; Edwards et al., 2008; Kingston-Smith et al., 2008, 2010). Once in soil, a portion of the N can be converted by bacteria into N_2_O, a GHG with a 298-fold greater global warming potential than CO_2_ (Hristov et al., 2013).

In summary, the rumen microbiome is central to addressing the grand challenges facing agriculture globally. A better understanding of the roles played by the constituent microbes is central to the development of advanced methods to manipulate the rumen microbiome in a manner that improves ruminant production whilst reducing environmental impact (Yáñez-Ruiz et al., 2015).

## The rumen microbiome

The rumen is a complex, dynamic ecosystem composed of mainly anaerobic bacteria, protozoa, anaerobic fungi, methanogenic archaea and phages. These microbes interact with each other and have a symbiotic relationship with the host, providing energy from the breakdown of plant cell wall carbohydrates that are largely inedible by humans (Mizrahi, 2013). Recently, it has also been hypothesized that these microbes display niche specialization in terms of nutrient utilization and they also engineer the rumen ecosystem in terms of subsequent microbial colonization and nutrient utilization (Pereira and Berry, 2017; Shaani et al., 2018). As a consequence of their highly evolved rumen microbiome, ruminants provide human-edible nutritious foods derived from marginal land, without competing with food crop production (Kingston-Smith et al., 2010).

### Rumen bacteria

The seminal work of Robert Hungate, the father of rumen microbiology, resulted in many of the culture technologies for anaerobic bacteria that are still widely used throughout the world (Hungate, 1966). These cultivation techniques enabled researchers to show that the rumen bacteria are the most abundant and diverse group of microorganisms in the rumen ecosystem. As a whole, they possess a multitude of enzymatic activities (i.e., amylases, cellulases, proteases, lipases) that carry out digestion of starch, plant cell walls, proteins and lipids in the rumen. Whilst there have been significant technological advancements during the last decade, the function of the rumen bacteria and their interactions with other members of the rumen microbiome is still poorly understood and consequently there are only a few examples where direct manipulation of the composition of this community has generated beneficial outcomes.

One of these successes relates to *Leucaena leucocephala*, which is a leguminous plant, that is high in protein and used as a ruminant feed in tropical countries. Nonetheless, the plant also produces toxins, causing salivation, live weight losses and generally poor animal performance. *L. leucocephala* contains the toxin mimosine which is converted in the rumen to 4-hydroxy-4(H)-pyridone (DHP), an effective goitrogen (Wallace, 2008) The rumen microbiomes of Hawaiian goats were shown to be tolerant to *L. leucocephala* (Jones and Megarrity, 1986) and further investigations revealed that these goats possessed a bacterium, *Synergistes jonesii* which was capable of degrading DHP. This is a unique example whereby understanding the role of the rumen bacteria transformed livestock nutrition, as *S. jonesii* is now used as an inoculum in many tropical countries as means of counteracting DHP toxicity (Wallace, 2008).

### Rumen archaea

**Figure 1:**
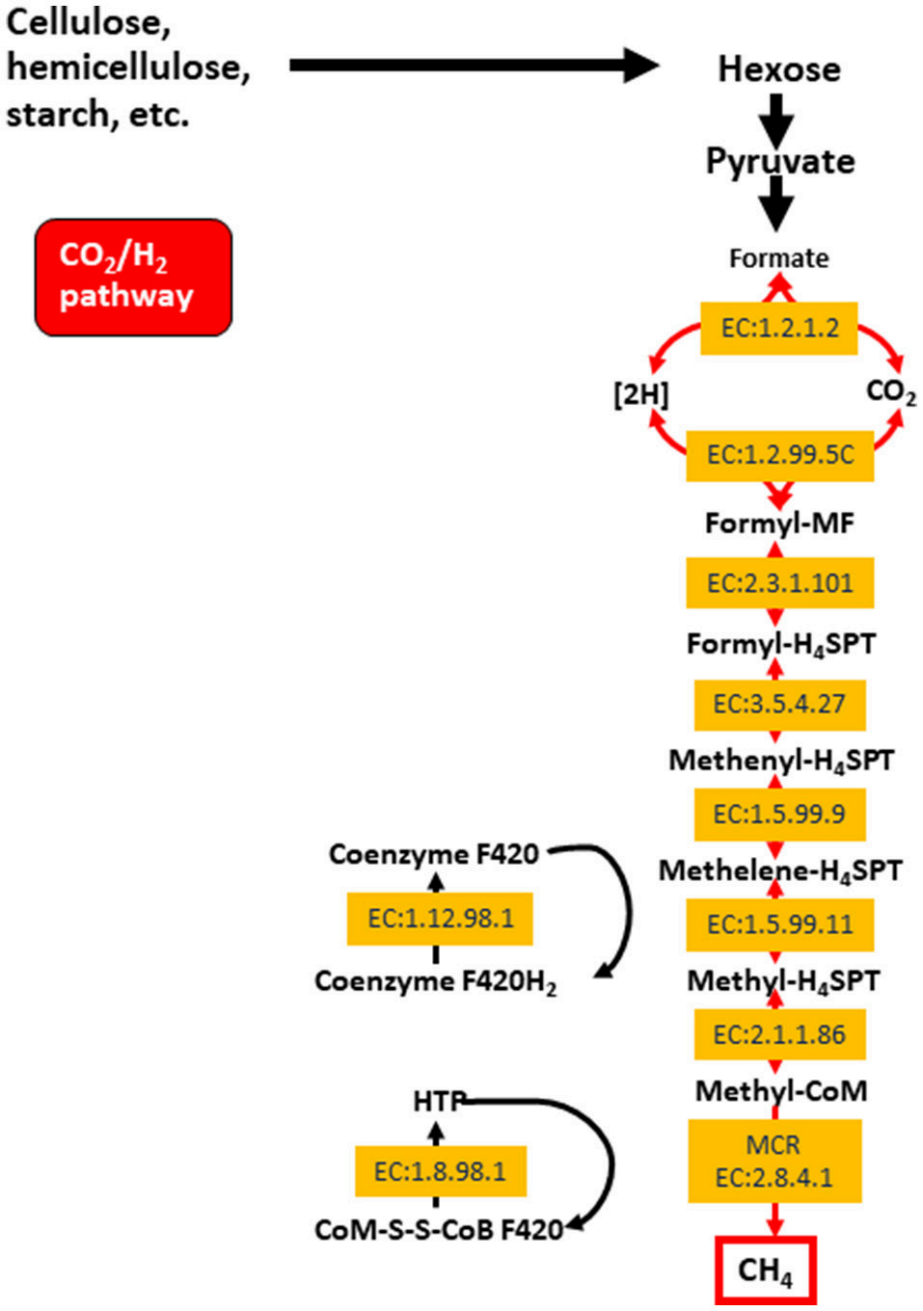
The hydrogenotrophic methane production pathway including enzyme classifications (EC) for enzyme involved in the process. Reproduced from Shi et al. (2014).

The archaeal domain in the rumen is composed largely of methanogenic archaea from the phylum Euryarchaeota. These methanogens are responsible for methane production in the rumen, which is then eructed and released to the environment. Methane is produced primarily via the hydrogenotrophic pathway (Figure 1) as a result of the reduction of CO_2_, and less so through the utilization of methyl groups (methylotrophic pathway), or even less commonly from acetate (acetoclastic pathway; Morgavi et al., 2010; Tapio et al., 2017). Hydrogenotrophic methanogens include *Methanobrevibacter (Mbb.)*, which is sub-divided into the SMT clade (*Mbb. smithii, Mbb. gottschalki, Mbb. millerae*, and *Mbb. thaurei*) or the RO clade (*Mbb. ruminantium* and *Mbb. Olleyae*; Tapio et al., 2017). Methylotrophic methanogens are less abundant and include *Methanosarcinales, Methanosphaera*, and *Methanomassiliicoccaceae*. Recently, methylotrophic methanogens and their functionality were found to be highly enriched in young ruminants whilst being less abundant and showing decreased functionality in mature animals (Friedman et al., 2017). Nonetheless, some published data show that *Methanomassiliicoccaceae* can represent approximately 50–70% of the rumen archaea (Huang et al., 2016; Wang P. et al., 2016). The *Methanosarcinales* can also produce methane via the acetoclastic pathway (Morgavi et al., 2010). Whilst methanogenesis has major implications for the environment, it serves an important purpose of elimination fermentative hydrogen from the rumen (Wright and Klieve, 2011). Strategies to reduce methane emissions must therefore take into account the need to remove excess hydrogen rom the rumen.

### Bacteriophage

Lytic phages were first isolated from rumen fluid and the bacterial genera *Serratia* and *Streptococcus* as far back as 1966 (Adams et al., 1966). Whilst much research ensued to isolate phage in the 1970s and 1980s, only those with potential biotechnological applications were further characterized and retained in culture collections (Gilbert and Klieve, 2015). Recently, Gilbert et al. (2017) isolated and obtained complete genome sequences for lytic phages, belonging to the order Caudovirales, capable of infecting *Bacteroides, Ruminococcus*, and *Streptococcus*. Whilst it is known that phage alter the ecology and evolution of microbial communities (Koskella and Brockhurst, 2014), the effects of phage on the rumen microbiome remains to be determined.

### Rumen protozoa

Whilst the rumen bacteria are the most numerate, the rumen protozoa represent a large proportion of the microbial biomass within the rumen (approximately 20% and up to 50% in some conditions) due to their cell volume. Rumen protozoa were first described by Gruby and Delafond in 1843 (Gruby and Delafond, 1843) and, along with fungi, make up the rumen eukaryote members of the microbiota (Williams and Coleman, 1997; Newbold et al., 2015). Ciliates dominate in the rumen, with flagellates such as *Trichomonas* sp., *Monocecromonas* sp. and *Chilomastix* sp. occasionally seen, but in much lower densities (Williams and Coleman, 1997). Ruminants commonly harbor distinct protozoal populations from birth, with only minor changes in diversity throughout life, although the abundances of species fluctuate with changes in diet (Williams and Coleman, 1997). For example, *Dastrychia* and *Entodinium* were shown to be the predominant genera in rumen fluid taken from dairy cows and *Dastrychia* has been shown to be more predominant in the rumen fluid taken from cows fed corn stover as compared with those fed alfalfa hay and corn silage (Zhang et al., 2015). Protozoal populations in the rumen have also been categorized as A-type (characterized by an abundance of *Polyplastron multivesiculatum*), B-type (characterized by an abundance of *Epidinium caudatum* or *Eudiplodinium maggii*), O-type (characterized by an abundance of *Entodinum, Dasytrycha*, and *Isotricha*), or lastly K-type (characterized by an abundance of *Elytroplastron bubali*; Kittelmann and Janssen, 2011).

**Figure 2:**
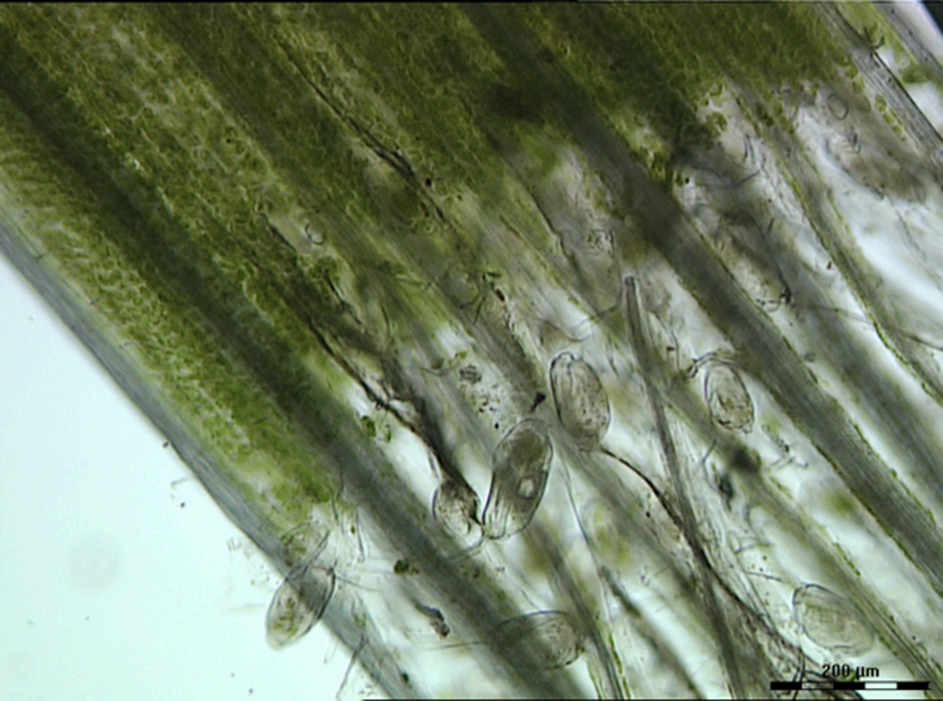
Light microscopy image of rumen contents taken from a ruminant possessing B-type protozoal diversity and showing close interactions of *Epidinium* spp. with fresh perennial ryegrass. Scale bar: 200 μM.

The contribution of protozoa to rumen fermentation remains controversial. It is known that protozoa can be removed from the rumen, a process known as defaunation, and the animal will still survive (Williams and Coleman, 1992; Newbold et al., 2015). A recent meta-analysis used 23 *in vivo* defaunation studies in an effort to determine the function of rumen protozoa (Newbold et al., 2015). Based on their analysis, Newbold and colleagues found evidence that the removal of protozoa from the rumen caused a decrease in organic matter degradation, especially of neutral and acid detergent fiber. This confirmed the original data of Williams and Coleman (1992) that some of the rumen protozoa (i.e., *Epidinium, Polyplastron* and *Entodinium* spp.) possess fibrolytic activity. Indeed, light microscopy of rumen contents clearly shows that *Epidinium* spp. are strongly associated with plant cells and are capable of scavenging plant chloroplasts, which are rich in protein and lipids (Huws et al., 2009, 2012; Figure 2). In addition to their capacity to degrade fiber, protozoa have been linked to methanogenesis as defaunation reduces methane output by approximately 11% (Hegarty, 1999; Morgavi et al., 2010; Newbold et al., 2015). This is likely due to the fact that rumen protozoal hydrogenosomes produce H_2_, which then serves as a substrate for methanogens to reduce CO_2_ to methane via the hydrogenotrophic pathway (Vogels et al., 1980; Belanche et al., 2014). This suggests that removal of protozoa may be a strategy to reduce production of methane by ruminants. However, rumen protozoa vary substantially in their contributions to plant degradation and methane production. For example, *Epidinium* spp. contribute substantially to plant degradation (Huws et al., 2009) and generally holotrichs support methanogens and methanogenesis (Belanche et al., 2014). As a consequence, a strategy which eliminates all protozoa may not be the best approach, nonetheless, elimination of a sub-group of protozoa is a major challenge which currently is technologically challenging.

### Rumen fungi

The flagellated zoospores of anaerobic fungi (Neocallimastigomycetes) were first observed in the early 1900's. However, it was not until the 1970's that their true identity was confirmed (Orpin, 1974, 1977a). To date, nine anaerobic fungal genera have been characterized with many other uncultivated taxa known to exist (Koetschan et al., 2014; Edwards et al., 2017; Paul et al., 2018). Paul et al. (2018) attempted to get consensus on the diversity of anaerobic fungi inhabiting the guts of herbivores and concluded that among the cultured genera, *Piromyces* was the most represented with *Buwchfawromyces* being the least represented in sequence data obtained from the Genbank database. Paul et al. (2018) also suggest that possibly another 25 new genera exist in the guts of herbivores, which remain uncharacterized. Irrespective, anaerobic fungi are among the most potent fiber-degrading organisms in the known biological world, primarily due to their efficient and extensive set of enzymes for the degradation of plant structural polymers (Solomon et al., 2016). Furthermore, their rhizoids have the ability to physically penetrate plant structural barriers (Orpin, 1977a,b). The latter ability benefits other rumen microbes by increasing the plant cell surface area available for colonization. Rumen fungi also possess amylolytic (Gordon and Phillips, 1998) and proteolytic activity (Gruninger et al., 2014).

The activity of anaerobic fungi is enhanced by methanogenic archaea (Cheng et al., 2009), which are known to physically attach to anaerobic fungal biomass. Anaerobic fungi are clearly beneficial, and have been shown to improve feed intake, feed digestibility, feed efficiency, daily weight gain and milk production (Lee et al., 2000; Dey et al., 2004; Paul et al., 2004; Tripathi et al., 2007; Saxena et al., 2010; Puniya et al., 2015). Chitin measurements (Rezaeian et al., 2004) and rRNA transcript abundance (Elekwachi et al., 2017) indicate that anaerobic fungi represent 10–20% of the rumen microbiome. However, like protozoa, they are not routinely studied despite suitable cultivation independent tools being available (Edwards et al., 2017).

Despite the importance of the rumen eukaryotes, our understanding of their function is far less than that of rumen bacteria. Beyond the study of their fiber degrading enzymes, much of the activity and metabolism of anaerobic fungi remains unknown, particularly due to the limited annotation of the multiple genome sequences and transcriptomes now available (Edwards et al., 2017). As with protozoa, key challenges include their cultivation, lack of genomic information, and lack of consensus on best practices to analyse sequence data (Ishaq et al., 2017). Thus, there are still many challenges which need to be overcome to enable a comprehensive understanding of the rumen microbiome as a whole.

## Importance of the biofilm phenotype and membrane vesicle production to host nutrient availability

**Figure 3:**
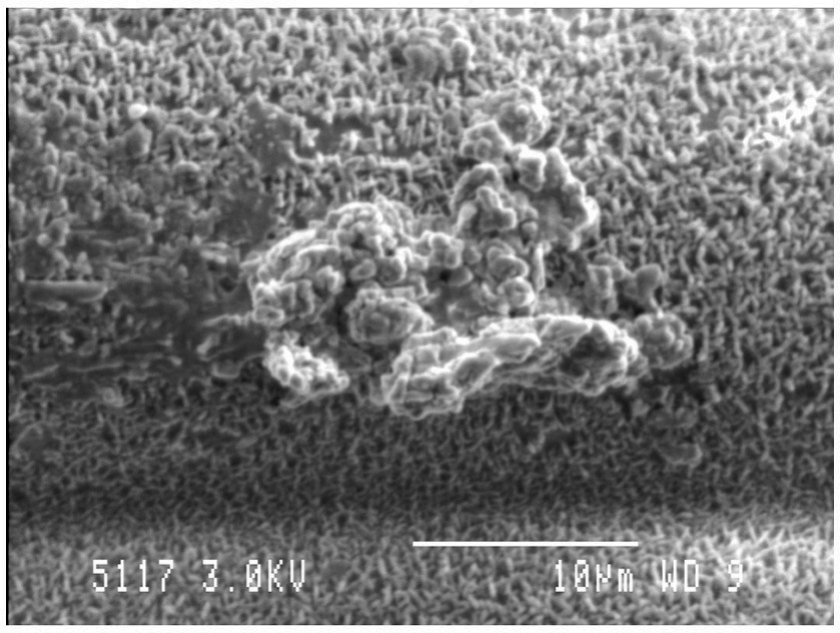
Biofilm community on the adaxial surface of fresh perennial following *in vitro* incubation in the presence of rumen fluid as outlined in Huws et al. (2014). Scale bar: 10 μM.

Similar to most other microbiomes in nature, the rumen microbiome is dominated by microbes existing within biofilms, which are defined as a consortia of microbes attached to a surface, encased in a self-produced extracellular polymeric matrix (EPS; Figure 3; Cheng et al., 1979; Cheng and Costerton, 1980; Mcallister et al., 1994; Huws et al., 2013, 2014, 2016; Zhao et al., 2018). The biofilm phenotype has many advantages, including the concentration of digestive enzymes within the EPS in proximity to the substrate, an arrangement that enables effective hydrolysis of plant material within the rumen (Minato et al., 1966; Wolin et al., 1997; Michalet-Doreau et al., 2001; Leng, 2014). The EPS is also rich in DNA, protein, and lipids, which possibly play a role in biofilm stability, whilst also being a source of nutrients for the ruminant following its out-flow from the rumen to the lower digestive tract (Shukla and Rao, 2017; Sugimoto et al., 2018). Whilst protein concentration within EPS is greater than within the attached bacteria, very little consideration has been given to this structure in terms of contribution to the nutrition of the host.

**Figure 4:**
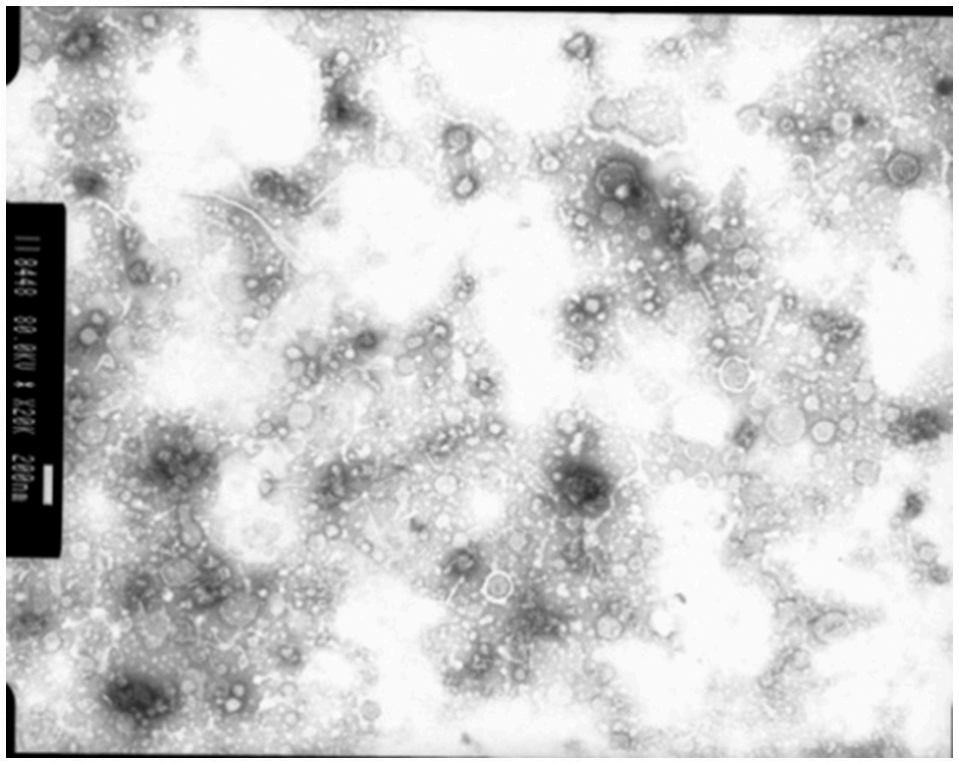
Membrane vesicles isolated from *Prevotella ruminocola* incubated *in vitro* in Hungate tubes. Scale bar: 200 nM.

Membrane vesicles are often blebbed from the bacterial cell membrane, that extend into the EPS. Numerous bacterial pure culture studies have shown that bacteria are adept at producing membrane vesicles (Schooling and Beveridge, 2006). These membrane vesicles are packed with DNA, proteins and lipids (Schooling et al., 2009) and likely promote biofilm stability. These membrane vesicles have been recently observed in the rumen bacterium *Fibrobacter succinogenes* (Arntzen et al., 2017). These membrane vesicles can contain high concentrations of glycosyl hydrolases, allowing *F. succinogenes* to effectively degrade plant cellulose (Arntzen et al., 2017). Also, *Prevotella ruminocola* is suggested to produce membrane vesicles, but their role in plant degradation remains to be defined (Huws, personal communication; Figure 4).

## Untangling the influence of diet on the rumen microbiome and consequently host phenotype

### Adult animals

A recent global comparison study of the rumen microbiome in 742 samples across 32 species from various geographical locations (Henderson et al., 2015), identified that 30 of the most abundant bacterial groups were present in over 90% of the samples. Members of the methanogen clades *Methanobrevibacter gottschalkii* and *Methanobrevibacter ruminantium* were found in nearly all samples and accounted for 74% of the archaea. The consistency of common microbes across a wide variety of ruminants led Henderson et al. (2015) to conclude that global evolutionary pressures selected for common microbial components within the fermentatative microbiomes. This reasons with Darwin's theory of natural selection, considering a natural diet high in forage is common amongst ruminants. This study also concluded that the composition of the rumen microbiome was mainly driven by the diet (Henderson et al., 2015). Indeed, dietary interventions have been historically used to improve upon ruminant phenotypes due to their influence on the rumen microbiome (Table 1).

**Table 1:** Examples of dietary interventions that have been used to modulate the rumen microbiome.

**Dietary intervention**	**Effects**	**Mode of action**	**References**
High quality forage	Improve milk fat content and quality	Favor fibrolytic microbes and microbial diversity (resilience)	Couvreur et al., 2006
High concentrate diet	Increase animal productivity	Favor propionate production and decrease microbial diversity	Fernando et al., 2010; Belanche et al., 2012
Antibiotics	Increase productivity and decrease rumen acidosis	Favor propionate-producers	Schelling, 1984
Red Clover	Increase productivity	Protein complexing with polyphenol oxidase allowing more protein to by-pass rumen	Broderick, 1995; Lee, 2014; Hart et al., 2016
Bicarbonate	Prevention rumen acidosis	Limit the rumen pH depression and the negative impact on rumen microbes	Keunen et al., 2003; González et al., 2012
Probiotics (Yeast)	Prevention rumen acidosis and increase feed efficiency	Oxygen scavenging	Newbold et al., 1996; Desnoyers et al., 2009
Essential oils	Prevention rumen acidosis	Shift in the microbial community	Calsamiglia et al., 2007; Macheboeuf et al., 2008
Tannins	Improve the flow of digestible rumen by-pass protein	Formation of phenol-dietary protein complexes	Mcsweeney et al., 2001; Patra, 2010
Saponins	Methane inhibition and increased microbial protein synthesis	Antiprotozoal effect	Patra and Saxena, 2009b; Ramos-Morales et al., 2017
Unsaturated fats	Methane inhibition	Antiprotozoal effect	Martin et al., 2010
Methane analogs	Methane inhibition	Inhibition of rumen methanogens	Knight et al., 2011; Abecia et al., 2012; Hristov et al., 2015

Among these dietary interventions, the modification of the forage:concentrate ratio is the most studied (Fernando et al., 2010). Ruminants have traditionally been fed high forage diets to decrease feeding costs, and to avoid competition with plant sources that can be used as food for humans. Moreover, a linear relationship has been noted between the proportion of fresh grass within the diet, and milk fat composition and butter properties in dairy cattle (Couvreur et al., 2006). In particular, fresh grass in comparison to grass hay promotes an accelerated feed colonization by rumen microbes and subsequent digestion (Belanche et al., 2017). Furthermore, microbial protein synthesis is increased and methane emissions lowered (Belanche et al., 2016). However, most of the intensive ruminant production systems, particularly beef feedlot systems, use high-grain diets to maximize growth rates and feed efficiency. Supplementation of the diet with easily digestible carbohydrates minimizes the negative effects of dietary protein shortage (Belanche et al., 2012) and promotes a modification of the rumen microbiome (Fernando et al., 2010), due to a simplification of the rumen microbial community. As a result, animals fed high-grain diets tend to have lower bacterial diversity and lower concentrations of fibrolytic microbes (i.e., protozoa and anaerobic fungi), which are generally associated with lower rumen proteolysis and ultimately higher feed efficiency (Belanche et al., 2012). Moreover, high grain diet was shown to affect the composition of the rumen methanogenic community via its effect on the rumen redox potential, with a specific effect on the Methanomicrobiales order (Friedman et al., 2017). However, this strategy often leads to a decrease in rumen pH due to high VFA and lactate accumulation and ultimately to digestive disorders (rumen acidosis with lactate accumulation occurring in severe cases only) and energy spilling reactions (Russell and Strobel, 1993). To prevent lactic acidosis, antibiotics such as ionophores, which select against Gram positive bacteria that produce lactate are often included in high-grain diets. However, globally antibiotics and growth promoters have been drastically reduced in livestock production, with a ban enforced in the EU (Russell and Houlihan, 2003). Novel cost-effective strategies to modulate rumen microbial fermentation need to be identified.

Feeding red clover to ruminants results in increased nitrogen efficiency due to the fact that it possesses the enzyme polyphenol oxidase (PPO; Broderick, 1995; Lee, 2014). PPO is a copper metallo-protein that, in the presence of oxygen, catalyzes the oxidation of endogenous phenols to quinones (Lee et al., 2004). PPO protects plant protein from ruminal degradation, allowing intact protein to by-pass to the abomasum. The mechanism of protein protection seems to be related to the deactivation of plant proteases by the PPO enzyme as well as PPO mediated protein-quinone binding (Mayer and Harel, 1979; Lee, 2014). PPO is located in the chloroplast and until recently the substrate for activating PPO was thought to exist only in the plant vacuole. Recent data now indicates that PPO preferentially protects proteins within chloroplasts, suggesting that there are also PPO-activating substrates within chloroplasts (Hart et al., 2016; Boeckx et al., 2017). It is also known that feeding red clover silage alters rumen microbial diversity compared with a perennial ryegrass silage-based diet, which contributes to changes seen in animal phenotype when red clover is fed (Huws et al., 2010).

Sodium bicarbonate and yeast (*Saccharomyces cerevisiae*) supplementation both have shown some success in preventing sub-acute acidosis (SARA; Keunen et al., 2003; González et al., 2012; Ishaq et al., 2017). Due to its oxygen scavenging activity in the rumen (Newbold et al., 1996), *S. cerevisiae* can increase the density of fibrolytic bacteria and hence feed efficiency (Desnoyers et al., 2009). Ishaq et al. (2017) also showed that dairy cows with diet-induced SARA had a higher abundance of rumen fungi and lower abundances of rumen protozoa compared with healthy cows. Ishaq et al. (2017) then fed active dry yeast to the dairy cows with induced SARA and noted an increase in pH and rumen protozoal abundance. Moreover, in recent years, a variety of plant bioactive compounds, including saponins, essential oils, tannins and flavonoids have also been evaluated for their ability to modulate rumen microbial fermentation (Patra and Saxena, 2009a,b). Essential oils have been proven to slow down starch and protein degradation, decreasing the risk of acidosis while causing minor reductions in rumen methanogenesis (Calsamiglia et al., 2007). However, their application has been limited because of their adverse effects on fiber digestion and rumen fermentation (Macheboeuf et al., 2008). Phenolic compounds such as condensed and hydrolysable tannins can also have anti-nutritional effects due to their interaction with enzymes and their antimicrobial properties. However, if fed at the right level, it is well established that tannins protect dietary protein from degradation in the rumen without significantly affecting the efficiency of carbohydrate digestion (Mcsweeney et al., 2001). Additionally, it has been suggested that tannins may also decrease methanogenesis by inhibition of rumen protozoa, methanogens and, to a lesser extent, hydrogen-producing microbes (Patra, 2010). Saponins, a group of plant secondary compounds derived mainly from *Yucca shidigera* and *Quillaja saponaria*, have also shown potential for modifying rumen fermentation primarily through the inhibition of protozoa. Furthermore, saponins can decrease methane production by selectively targeting certain groups of rumen protozoa, methanogens, fungi and bacteria (Patra and Saxena, 2009b). However, the antiprotozoal effect of saponins is transitory as when saponins are deglycosylated to sapogenins by rumen microorganisms, they become inactive (Newbold et al., 1997). This presents a challenge for the practical application use of saponins in ruminant nutrition (Ramos-Morales et al., 2017).

One of the most promising compounds for reducing ruminal methanogenesis is 3-NOP, which is an analog of the methyl-coenzyme M subunit of the nickel enzyme methyl-coenzyme M reductase in rumen archaea. This enzyme catalyzes the last step of methane-forming reactions (Duin et al., 2016) and its inhibition can result in a reduction in rumen methanogenesis (up to 30%) without negative effects to the animal (Hristov et al., 2015). Nonetheless, benefits for ruminant production are comparatively low, likely due to high H_2_ accumulation (Jayanegara et al., 2018). However, a recent study has shown that benefits for animal productivity could be enhanced (Martinez-Fernandez et al., 2017). Supplementation of phloroglucinol together with 3-NOP promotes capture of excess hydrogen from methanogenesis and generates valuable metabolites for the host (Martinez-Fernandez et al., 2017). The addition of acetogenic rumen bacteria to remove excess hydrogen has also been widely suggested as an effective intervention which may work in combination with 3-NOP (Wright and Klieve, 2011)

In summary, more multidisciplinary studies are needed to uncover the mode of action of these nutritional interventions and their true potential to modulate the rumen microbiome under farm conditions. Furthermore, data shows that in mature ruminants, dietary changes can be short-lived. Instead, interventions in the early life of the ruminant may offer a better longer-term strategy to improve animal phenotype.

### Early-life

In contrast to the developed rumen, where a stable and resilient microbial community is established, during the development of the rumen after birth a succession of different microbial groups colonize and start occupying the different ecological niches. The instability occurring during this period potentially allows for manipulation to assemble a specific community composition that persist later in life for better health and productivity within a given production system (Yáñez-Ruiz et al., 2015).

At birth, ruminants display a non-developed reticulo-rumen. Until the system is fully matured, they function as monogastrics, whereby the milk fed is not digested in the rumen but flows to the abomasum via an esophageal groove (Church, 1988). Colonization of the developing rumen begins immediately after birth and progresses through the first few months of life until a stable community establishes (Jami et al., 2013). The dynamics of the gut microbial community establishment in young ruminants occurs in three successive steps (Rey et al., 2014; Abecia et al., 2017): (i) initial colonization (0–2 days *post-partum*) originated from a combination of sources such as microbiota of mother's vagina, skin, colostrum and microbes within the environment (Van Nimwegen et al., 2011; Yeoman et al., 2018); (ii) transitional stage (3–15 days) during the transition from colostrum to milk, and iii) maturation stage in which solid feed intake progressively increases and the distribution of main bacterial phyla and other microbial groups is comparable to that in adult animals. It is important to note that although the rumen microbiome establishes before intake of solid feeds, the type of feed consumed plays a significant role in shaping the established rumen microbiome. Hence, the early phases of solid feed intake represents a window of opportunity to modulate the composition of the initial colonizers of the different ecological niches in the rumen according to dietary and management strategies (Yáñez-Ruiz et al., 2015). Indeed, the use of probiotics, such as lactic acid bacteria, in early life to mitigate incidence of digestive and respiratory diseases has shown promise (Timmerman et al., 2005; Signorini et al., 2012). Yáñez-Ruiz et al. (2010) also reported that feeding forage vs. concentrate around weaning modifies the bacterial population colonizing the rumen of lambs and that the effect persists over 4 months. It is also known that feeding concentrate in early life stimulates the development of the epithelium, while feeding high fiber diets can stimulate development of rumen muscularization and volume (Zitnan et al., 1998). Nonetheless, little is known regarding the impact of management practices, such as milk intake, delayed weaning etc. on early-life programming of the rumen microbiome and its implications for ruminant productivity.

Another factor that promotes differences in rumen colonization is the presence of the dam and the associated increase in the availability of microorganisms in the environment. This can allow earlier (and different) inoculation of microbes in the digestive tract of naturally raised newborns as compared to those fed milk replacer and kept in isolation (Abecia et al., 2017). Direct contact with the mother offers a constant source of microbes through the mouth, feces, skin and milk (Yeoman et al., 2018), sources that are not available for calves raised in isolation on milk replacer. This explains the greater number of Operational Taxonomic Units (OTUs) and bacterial diversity observed in naturally reared calves. Another distinctive feature between natural and artificial rearing systems is the near absence of protozoa in the rumen of artificially reared calves, as protozoa can only be inoculated in the rumen by direct contact with the dam or other mature animals through saliva (Abecia et al., 2014). A relatively recent study by Ishaq et al. (2015) showed that exposure of neonate lambs to the dam for 1 week followed by subsequent separation was enough to ensure the establishment of a stable rumen protozoal population for their lifetime.

Nutritional interventions in early-life may include (i) the direct inoculation of specific microorganisms or (ii) the use of additives that prevent or facilitate the colonization of some microbial groups. Feeding live microorganisms to ruminants is not a novel concept and extensive work has been published on the use of “direct-fed microbials” (DFM; Martin and Nisbet, 1992; Jeyanathan et al., 2014). The effect of supplementing *S. cerevisiae* on rumen development and growth performance in neonatal dairy calves has also been evaluated (Lesmeister et al., 2004). Although yeast cultures are widely used in ruminant nutrition, the concept of applying them in the diet of pre-ruminants deserves further assessment, especially in terms of their long term effects on the microbiome (Alugongo et al., 2017). A different approach that uses compounds to inhibit the establishment of certain microbial groups or favor the development of others is also now starting to attract attention. It has been shown that application of bromochloromethane (BCM) to young goat kids modified archaeal colonization of the rumen, and was linked to a reduction in methane emission of around 50%, with the effects persisting for 3 months after weaning (Abecia et al., 2013, 2014).

Despite some promising results from early-life dietary interventions, the ecological dynamics underpinning the microbial colonization, the most effective window of time for intervention and the long-term implications have yet to be identified.

## Untangling the influence of host genomics on the rumen microbiome and consequently host phenotype

Consistent with human twin heritability studies (Goodrich et al., 2016), it is reasonable to hypothesize that animals possessing similar genomes should have more similar rumen microbiomes. Evidence of the influence of the host on the rumen microbiome was first postulated by Weimer et al. (2010) who found that after near total exchange of the rumen contents between cows, individuals restored their bacterial composition back to pre-exchange conditions, which also returned rumen pH and volatile fatty acid (VFA) concentration to pre-exchange values. Furthermore, in another near-total rumen content exchange between high- and low-efficiency Holstein cows, Weimer et al. (2017) demonstrated the hosts ability to return the rumen bacterial community to the original status, whilst linking the rumen microbiome to milk production efficiency.

Whilst Henderson et al. (2015) postulated that diet was the main driver for rumen microbiome composition, they also identified some differences in the relative abundance of certain bacterial populations across ruminant species. Similarly, when the microbiome of water buffalo (*Bubalus bubalis*) and Jersey cows were compared under comparable feeding conditions variations in bacterial, protozoa and methanogen populations were found between the two species (Iqbal et al., 2018), suggesting that the rumen microbiome is controlled, to a certain extent, by the genetics of the host. In a beef cattle experiment, Roehe et al. (2016) ranked beef sire progeny groups based on relative archaeal abundance and reported that group ranking remained consistent overall and within diet, suggesting that archaeal abundance in ruminal digesta is also, in part under host genetic control. Using sire progeny groups in dairy cattle, further evidence of genetic control was documented by the discovery that 22 bacterial OTUs, exhibited a heritability estimate of 0.7 or greater in dairy cattle (Sasson et al., 2017). In addition, these heritable OTUs were found to be correlated with traits such as DMI (dry matter intake) and RFI. Pinares-Patiño et al. (2011) and Pinares-Patiño et al. (2013) demonstrated that methane production is also regulated by host genetics in sheep and that selection of low methane emitting animals by genotyping is possible.

Nonetheless, De Mulder et al. (2018), stated that the differences in rumen microbiome composition may be due to other factors other than host genomics, including early life events and the fact that some breeds of cattle, such as Belgian Blue cattle, have a higher rate of cesarean section birth. The host immune system also likely plays an influential role on the rumen microbiome. For example, secretory immunoglobulin A (SIgA), which favors commensal bacteria in the gut (Gutzeit et al., 2014), has been shown to coat rumen bacteria (Fouhse et al., 2017) and control the host's recognition of certain microbial species. In addition, the rumen epithelium plays an important role in both nutrient uptake and immunity. The physiology of the rumen has also been highlighted as a potential factor that influences the rumen microbiome. For example, differences in rumen and camelid foregut volume, physiology as well as feeding frequencies, was suggested as a reason for the proportionally higher abundance of unclassified *Veillonellaceae* in camelids, deer and sheep compared to cattle (Henderson et al., 2015). In addition, methane yield is associated with retention time in the rumen (Pinares-Patiño et al., 2003) correlating increased passage rate in the rumen with reduced methane yield. Janssen (2010) provides a thorough review of these studies which in essence show that increased passage rate leads to less feed being fermented in the rumen and subsequently less substrate is available for methanogenesis (Tapio et al., 2017). It has also been demonstrated that both a shorter rumen retention time and a smaller rumen result in reduced methane yield (Goopy et al., 2014). Additionally, variation in the rumination behavior of animals can influence particle retention time (Mcsweeney et al., 1989). Therefore, genetic influence of the host on rumen passage rate is likely to be one host factor that influences the rumen microbiome, but other factors should also be considered (Pinares-Patiño et al., 2013).

Whilst there is increased evidence that host genetics has an influential role on the microbial population residing in the rumen (Tapio et al., 2017), our current understanding of the extent of this influence and the underlying mechanisms (Sasson et al., 2017) remains incomplete, although a region on chromosome 6 was recently associated with Actinobacteria, Euryarchaeota, and Fibrobacteres densities (Golder et al., 2018).

## Contributions of the lower gastrointestinal tract microbiomes to ruminant phenotype

Typically, scientists have focussed their attention on understanding the rumen in order to deliver upon global livestock challenges. However, the lower gastrointestinal (GI) tract microbiomes also play an important role, particularly in early life (Meale et al., 2017). The microbial composition of the post-ruminal gastrointestinal tract is shaped by pH, gut motility, redox potential, and host secretions present in different compartments of the digestive tract. Most microbes flowing from the rumen into the abomasum are lysed by the low pH and enzymatic activity within the organ. As a consequence of the harsh environmental conditions prevailing in the abomasum and at the beginning of the small intestine, microbial numbers and diversity plummet by several orders of magnitude in the abomasum, duodenum and jejunum as compared to the rumen (Frey et al., 2010; He et al., 2018; Yeoman et al., 2018). From the ileum onwards, including caecum, colon and feces, favorable fermentation conditions are present again and microbial density and phylogenetic diversity increase to a level comparable to that of the rumen (Frey et al., 2010; De Oliveira et al., 2013; Popova et al., 2017; He et al., 2018; Yeoman et al., 2018).

The post-ruminal microbial community is composed predominantly of bacteria, but methanogenic archaea and anaerobic fungi have been described (Davies et al., 1993), although the later phylogentic group has not been targeted intensively with high-throughput sequencing techniques. There are significant difference in the microbial community assemblage depending on the region of the GI tract (i.e., rumen vs. post-rumen; Mao et al., 2015; Bergmann, 2017; Zeng et al., 2017; Yeoman et al., 2018), and the post rumen microbiota differ further between the small (duodenum, jejunum, and ileum) and the large (cecum, colon, and rectum) intestine (Mao et al., 2015; Bergmann, 2017; Wang et al., 2017; Yeoman et al., 2018). In general terms, compared to the rumen, the proportion of Bacteroidetes decrease and that of Firmicutes and Proteobacteria increase. *Prevotella, Bacteroides, Ruminococcus, Treponema*, and *Desulfovibrio* genera were detected in all segments of the GI tract of ruminant animals, while *Fibrobacter* was only present in the foregut (Zeng et al., 2017). *Prevotella, Bacteroides, Ruminococcus, Faecalibacterium, Roseburia* and *Clostridium* are consistently identified in fecal samples from ruminants and are considered part of the core microbiota (Dowd et al., 2008; Durso et al., 2012). As for the rumen, the rectal microbiota shows important inter-individual variation (Durso et al., 2010) and are affected by diet (Shanks et al., 2011).

The mucosa-associated microbial community is also an important modulator of immunological function and health (Malmuthuge et al., 2015). Mucosa-associated communities differ from those associated with luminal contents; and also vary among intestinal regions (Malmuthuge et al., 2014; Mao et al., 2015; Yeoman et al., 2018). Potential pathogens such as *Escherichia, Shigella, Salmonella* and *Treponema* spp. are most frequently found in the mucosa-associated bacterial microbiota (Mao et al., 2015; Song et al., 2018). Recently, differences in both the mucosa-associated microbiota of the rectoanal junction and fecal microbiota of cattle have been shown to influence the shedding of the human pathogen *Escherichia coli* O157 in cattle feces (Stenkamp-Strahm et al., 2018; Wang et al., 2018).

The role of the intestinal microbiota in feed degradation appears to be less important than that of the rumen (Al-Masaudi et al., 2017). Its main function has been suggested to be related to animal health and cross-talk interaction with the animal host (Lyte et al., 2018), although work in this area is only in its nascent phase and these aspects need further investigation. Notwithstanding, it is highlighted that feces and samples from the intestines cannot be used as proxies of rumen function on a microbiome biomarker level (Tapio et al., 2016). Nonetheless, concentrations of the compound archaeol in feces has been shown to correlate with methane emissions in cattle (Mccartney et al., 2014).

## Developing microbiome biomarkers for prediction of ruminant phenotype

The sheer size of the rumen (12–15% of body mass) and connectedness with the vascular, respiratory and immune systems mean that it is well-placed to both affect, and be affected by, animal function. There is a growing number of examples where the interaction between host and intestinal microbial metabolism can be used to explain, or act as a biomarker for, complex traits such as nutrient efficiency, responses to stressors such as disease and adverse environments, as well as to predict animal behavior.

Nucleic acids have long been used as biomarkers for rumen microbial processes. Early work focussed on rumen microbial protein synthesis and RNA (Mcallan and Smith, 1969), while purine bases (Zinn and Owens, 1986) were also used as biomarkers for microbial (protein) synthesis in studies with intestinally cannulated animals. More recent attempts to develop non-invasive biomarker approaches to estimate microbial protein synthesis have used urinary metabolites derived from microbial purines (allantoin and uric acid; Chen et al., 1990). Recent advances in analytical technologies and bioinformatics have now greatly expanded our capacity to investigate the role of the rumen and its microbiome in complex traits by studying the composition of microbial DNA and RNA (metataxonomics, metagenomics and metatranscriptomics), as well as microbial metabolites in blood or urine (metabolomics). In terms of metataxonomics, microbial correlations to feed efficiency and/or methane production in ruminants, using rRNA genes or the Methyl coenzyme M reductase (mcrA) gene in methanogens are difficult to interpret, due to the confounding factors such as animal type, feed, and rumen sample processing and analysis (see Metataxonomy section). Recent studies suggest that methanogen diversity, and not density, is critically important to methane output, with more diversity being associated with higher emissions (Janssen and Kirs, 2008; Carberry et al., 2014). However, most studies involve a small number of animals, making it difficult to clearly confirm the link between methanogen diversity and methane emissions (Morgavi et al., 2010). When investigating the rumen bacterial associations with methane production, density of *Sharpea* has been shown to be significantly lower in low methane emitting animals (Kamke et al., 2016). Positive correlations between *Eubacterium* sp. and reduced feed efficiency were also reported by Hernandez-Sanabria et al. (2012). Jami et al. (2014) also reported a positive correlation between RFI and the uncultured rumen bacterium RF39, whereas Shabat et al. (2016), suggested that an increase in the acrylate pathway coded by *Megasphaera elsdenii* and *Coprococcus catus* in the rumen may increase feed efficiency and reduce methane. It has been suggested also that the ratio of bacteria:archaea reflects methane output from the animal with positive correlations reported in a few studies (Wallace et al., 2014; Auffret et al., 2017b), but results are not consistent (Tapio et al., 2017). Recent data also suggest that the rumen microbiome of feed efficient ruminants is less diverse than their inefficient counterparts (Shabat et al., 2016; Li and Guan, 2017). The microbial diversity within the rumen offers the animal resilience from dietary related perturbations, such as acidosis. Therefore, care must be taken to ensure that breeding for increased feed efficiency in ruminants does not negatively impact resilience of the microbiome and increase the susceptibility of the host to digestive diseases. Irrespective, metataxonomic data is highly variable due largely to the differences in techniques employed across published datasets (see Metataxonomy section) and animal variation. As such the use of gene biomarkers using metagenomics and/or metranscriptomic approaches may be more useful given that rumen microbes possess genes coding for a high level of functional redundancy (Edwards et al., 2008; Weimer, 2015).

Recent work using metagenomics and/or metatranscriptomics has confirmed significant relationships between the abundances of key rumen microbial genes and feed efficiency (Roehe et al., 2016; Shabat et al., 2016; Li and Guan, 2017) and/or methane production (Roehe et al., 2016). Due to the vastness of these datasets it is difficult to compare and investigate whether studies commonly find consensus genes that would serve as good global biomarkers in their correlation studies. Microbial gene correlations with RFI, data from Shabat et al. (2016) and Li and Guan (2017) showed some consensus as both showed that genes involved in amino acid metabolism were less abundant in feed efficient animals (Table 2). These data corresponds with observations that feed efficient animals excrete less urinary ammonia suggesting better rumen nitrogen use efficiency (Bach et al., 2005; Broderick and Reynal, 2009). Consensus of other genes across the three published datasets were not found (Table 3). Likewise, genes correlating to methane emissions show very little consensus amongst the five papers investigated. Nevertheless, in four datasets the methyl coenzyme reductase enzyme, which is involved in the last step of the hydrogenotrophic methane pathway (Figure 1), showed the most correlation to methane. The lack of consensus across experiments, whilst perhaps being biologically correct, likely also reflects the challenges associated with comparing of datasets from different animals, variation in diet, as well as differences in sampling method, sample preparations and data interpretation. Clearly more comparative large datasets are required to develop microbiome based biomarkers for estimation of RFI and methane output. Alongside this is the need to obtain samples that are representative of the rumen microbiome in a non-invasive manner. Recently it was suggested that the oral microbiome of ruminants reflects the microbial diversity seen in the rumen (Tapio et al., 2016), raising the possibility that buccal swabbing may be used as a proxy for rumen samples.

**Table 2:** Potential gene biomarkers indicative of feed efficiency in ruminants.

**KEGG number**	**Description**	**E.C number**	**Pathway**	**Published papers**
K00075	UDP-N-acetylmuramate dehydrogenase	EC:1.3.1.98	Amino acid sugar and nucleotide sugar metabolism; Peptidoglycan biosynthesis; Metabolic pathways	Roehe et al., 2016
K00121	S-(hydroxymethyl)glutathione dehydrogenase/alcohol dehydrogenase	EC:1.1.1.284 or 1.1.1.1	Glycolysis/Gluconeogenesis; Fatty acid degradation; Tyrosine metabolism; Chloroalkane and chloroalkene degradation; Naphthalene degradation; Methane metabolism; Retinol metabolism; Metabolism of xenobiotics by cytochrome P450; Drug metabolism–cytochrome P450; Metabolic pathways; Biosynthesis of secondary metabolites; Microbial metabolism in diverse environments; Biosynthesis of antibiotics; Carbon metabolism; Degradation of aromatic compounds; Chemical carcinogenesis	Li and Guan, 2017
K00179	Indolepyruvate ferredoxin oxidoreductase, alpha subunit	EC:1.2.7.8	-	Roehe et al., 2016
K00230	Menaquinone-dependent protoporphyrinogen oxidase	EC:1.3.5.3	Porphyrin and chlorophyll metabolism; Metabolic pathways; Biosynthesis of secondary metabolites	Li and Guan, 2017
K00240	Succinate dehydrogenase/fumarate reductase, iron-sulfur subunit	EC:1.3.5.1 or 1.3.5.4	Citrate cycle (TCA cycle); Oxidative phosphorylation; Butanoate metabolism; Carbon fixation pathways in prokaryotes; Metabolic pathways; Biosynthesis of secondary metabolites; Microbial metabolism in diverse environments; Biosynthesis of antibiotics; Carbon metabolism	Li and Guan, 2017
K00260	Glutamate dehydrogenase	EC:1.4.1.2	Arginine biosynthesis; Alanine, aspartate and glutamate metabolism; Taurine and hypotaurine metabolism; Nitrogen metabolism; Metabolic pathways	Shabat et al., 2016
K00270	Phenylalanine dehydrogenase	EC:1.4.1.20	Tyrosine metabolism; Phenylalanine metabolism; Phenylalanine, tyrosine and tryptophan biosynthesis; Metabolic pathways; Biosynthesis of secondary metabolites; Biosynthesis of antibiotics	Shabat et al., 2016; Li and Guan, 2017
K00278	L-aspartate oxidase	EC:1.4.3.16	Alanine, aspartate and glutamate metabolism; Nicotinate and nicotinamide metabolism; Metabolic pathways	Roehe et al., 2016
K00281	Glycine dehydrogenase	EC:1.4.4.2	Glycine, serine and threonine metabolism; Glyoxylate and dicarboxylate metabolism; Metabolic pathways; Biosynthesis of secondary metabolites; Biosynthesis of antibiotics; Carbon metabolism	Li and Guan, 2017
K00290	Saccharopine dehydrogenase (NAD+, L-lysine forming)	EC:1.5.1.7	Lysine biosynthesis; Lysine degradation; Metabolic pathways; Biosynthesis of secondary metabolites; Biosynthesis of antibiotics; Biosynthesis of amino acids	Shabat et al., 2016
K00315	Dimethylglycine dehydrogenase	EC:1.5.8.4	Glycine, serine and threonine metabolism; Metabolic pathways	Li and Guan, 2017
K00330	NADH-quinone oxidoreductase subunit A	EC:1.6.5.3	Oxidative phosphorylation; Metabolic pathways	Shabat et al., 2016
K00340	NADH-quinone oxidoreductase subunit K	EC:1.6.5.3	as above	Shabat et al., 2016; Li and Guan, 2017
K00350	Na+-transporting NADH:ubiquinone oxidoreductase subunit E	EC:1.6.5.8	-	Shabat et al., 2016; Li and Guan, 2017
K00360	Assimilatory nitrate reductase electron transfer subunit	EC:1.7.99.-	Nitrogen metabolism; Microbial metabolism in diverse environments	Shabat et al., 2016
K00362	Nitrite reductase (NADH) large subunit	EC:1.7.1.15	Nitrogen metabolism; Microbial metabolism in diverse environments	Li and Guan, 2017
K00375	GntR family transcriptional regulator/MocR family aminotransferase	-	-	Roehe et al., 2016
K00380	Sulfite reductase (NADPH) flavoprotein alpha-component	EC:1.8.1.2	Sulfur metabolism; Metabolic pathways; Microbial metabolism in diverse environments	Shabat et al., 2016
K00394	Adenylylsulfate reductase, subunit A	EC:1.8.99.2	Sulfur metabolism; Metabolic pathways; Microbial metabolism in diverse environments	Roehe et al., 2016
K00400	Methyl coenzyme M reductase system, component A2	-	Methane metabolism; Metabolic pathways; Microbial metabolism in diverse environments	Shabat et al., 2016
K00471	Gamma-butyrobetaine dioxygenase	EC:1.14.11.1	Lysine degradation	Li and Guan, 2017
K00480	Salicylate hydroxylase	EC:1.14.13.1	Dioxin degradation; Polycyclic aromatic hydrocarbon degradation; Naphthalene degradation; Metabolic pathways; Microbial metabolism in diverse environments; Degradation of aromatic compounds	Li and Guan, 2017
K00520	Mercuric reductase	EC:1.16.1.1	-	Li and Guan, 2017
K00521	Ferric-chelate reductase	EC:1.16.1.7	-	Li and Guan, 2017
K00633	Galactoside O-acetyltransferase	EC:2.3.1.18	-	Li and Guan, 2017
K00670	N-alpha-acetyltransferase 30	EC:2.3.1.256	-	Li and Guan, 2017
K00680	Uncharacterized N-acetyltransferase	EC:2.3.1.-	-	Shabat et al., 2016
K00730	Oligosaccharyl transferase complex subunit OST4	-	N-Glycan biosynthesis; Various types of N-glycan biosynthesis; Metabolic pathways; Protein processing in endoplasmic reticulum	Li and Guan, 2017
K00766	Anthranilate phosphoribosyltransferase	EC:2.4.2.18	Phenylalanine, tyrosine and tryptophan biosynthesis; Metabolic pathways; Biosynthesis of secondary metabolites; Biosynthesis of antibiotics; Biosynthesis of amino acids	Roehe et al., 2016
K00770	1,4-beta-D-xylan synthase	EC:2.4.2.24	Amino sugar and nucleotide sugar metabolism; Metabolic pathways	Li and Guan, 2017
K00785	Beta-galactosamide-alpha-2,3-sialyltransferase	EC:2.4.99.-	-	Li and Guan, 2017
K00790	UDP-N-acetylglucosamine 1-carboxyvinyltransferase	EC:2.5.1.7	Amino sugar and nucleotide sugar metabolism; Metabolic pathways; Peptidoglycan biosynthesis	Li and Guan, 2017
K00860	Adenylylsulfate kinase	EC:2.7.1.25	Purine metabolism; Sulfur metabolism; Metabolic pathways; Microbial metabolism in diverse environments	Li and Guan, 2017
K00868	Pyridoxine kinase	EC:2.7.1.35	Vitamin B6 metabolism; Metabolic pathways	Roehe et al., 2016
K00900	6-Phosphofructo-2-kinase	EC:2.7.1.105	Fructose and mannose metabolism	Li and Guan, 2017
K00908	Calcium/calmodulin-dependent protein kinase kinase 1	EC:2.7.11.17	Alcoholism	Li and Guan, 2017
K00920	1-Phosphatidylinositol-5-phosphate 4-kinase	EC:2.7.1.149	Inositol phosphate metabolism; Phosphatidylinositol signaling system; Regulation of actin cytoskeleton	Li and Guan, 2017
K00941	Hydroxymethylpyrimidine/phosphomethylpyrimidine kinase	EC:2.7.1.49 or 2.7.4.7	Thiamine metabolism; Metabolic pathways	Roehe et al., 2016
K00956	Sulfate adenylyltransferase subunit 1	EC:2.7.7.4	Purine metabolism; Monobactam biosynthesis; Selenocompound metabolism; Sulfur metabolism; Metabolic pathways; Microbial metabolism in diverse environments; Biosynthesis of antibiotics	Roehe et al., 2016
K00974	tRNA nucleotidyltransferase (CCA-adding enzyme)	EC:2.7.7.72 or 3.1.3.- 3.1.4.-	RNA transport	Roehe et al., 2016
K01051	Pectinesterase	EC:3.1.1.11	Pentose and glucuronate interconversions; Metabolic pathways	Li and Guan, 2017
K01055	3-Oxoadipate enol-lactonase	EC:3.1.1.24	Benzoate degradation; Metabolic pathways; Microbial metabolism in diverse environments; Degradation of aromatic compounds	Li and Guan, 2017
K01104	Protein-tyrorsine phosphatase	EC:3.1.3.48	-	Roehe et al., 2016
K01129	dGTPase	EC:3.1.5.1	Purine metabolism	Roehe et al., 2016
K01195	Beta-glucuronidase	EC:3.2.1.31	Pentose and glucuronate interconversions; Metabolic pathways; Glycosaminoglycan degradation; Porphyrin and chlorophyll metabolism; Flavone and flavonol biosynthesis; Drug metabolism–other enzymes; Biosynthesis of secondary metabolites; Lysosome	Roehe et al., 2016
K01269	Aminopeptidase	EC:3.4.11.-	-	Roehe et al., 2016
K01358	ATP-dependent Clp protease, protease subunit	EC:3.4.21.92	Cell cycle–Caulobacter; Longevity regulating pathway–worm	Roehe et al., 2016
K01493	dCMP deaminase	EC:3.5.4.12	Pyrimidine metabolism; Metabolic pathways	Roehe et al., 2016
K01613	Phosphatidylserine decarboxylase	EC:4.1.1.65	Glycerophospholipid metabolism; Metabolic pathways; Biosynthesis of secondary metabolites	Roehe et al., 2016
K01784	UDP-glucose 4-epimerase	EC:5.1.3.2	Galactose metabolism; Amino sugar and nucleotide sugar metabolism; Metabolic pathways	Roehe et al., 2016
K01814	Phosphoribosylformimino-5-aminoimidazole carboxamide ribotide isomerase	EC:5.3.1.16	Histidine metabolism; Metabolic pathways; Biosynthesis of secondary metabolites; Biosynthesis of amino acids	Roehe et al., 2016
K01818	L-fucose/D-arabinose isomerase	EC:5.3.1.25 or 5.3.1.3	Fructose and mannose metabolism; Microbial metabolism in diverse environments	Roehe et al., 2016
K01876	Aspartyl-tRNA synthetase	EC:6.1.1.12	Aminoacyl-tRNA biosynthesis	Roehe et al., 2016
K01924	UDP-N-acetylmuramate–alanine ligase	EC:6.3.2.8	D-Glutamine and D-glutamate metabolism; Peptidoglycan biosynthesis; Metabolic pathways	Roehe et al., 2016
K01928	UDP-N-acetylmuramoyl-L-alanyl-D-glutamate−2,6-diaminopimelate ligase	EC:6.3.2.13	Lysine biosynthesis; Peptidoglycan biosynthesis	Roehe et al., 2016
K02006	Obalt/nickel transport system ATP-binding protein	-	ABC transporters	Roehe et al., 2016
K02008	Cobalt/nickel transport system permease protein	-	ABC transporters	Roehe et al., 2016
K02030	Polar amino acid transport system substrate-binding protein	-	-	Li and Guan, 2017
K02313	Chromosomal replication initiator protein	-	Two-component system; Cell cycle–Caulobacter	Roehe et al., 2016
K02343	DNA polymerae III gamma/tau	EC:2.7.7.7	Purine metabolism; Pyrimidine metabolism; Metabolic pathways; DNA replication; Mismatch repair; Homologous recombination	Roehe et al., 2016
K02377	GDP-L-fucose synthase	EC:1.1.1.271	Fructose and mannose metabolism; Amino sugar and nucleotide sugar metabolism; Metabolic pathways	Roehe et al., 2016
K02907	Large subunit ribosomal protein L30	-	Ribosome	Roehe et al., 2016
K03111	Single-strand DNA binding protein	-	DNA replication; Mismatch repair; Homologous recombination	Roehe et al., 2016
K03410	Chemotaxis protein CheC	-	Bacterial chemotaxis	Li and Guan, 2017
K03426	NAD+ diphosphatase	EC:3.6.1.22	Nicotinate and nicotinamide metabolism; Metabolic pathways; Peroxisome	Roehe et al., 2016
K03458	Nucleobase:cation symporter-2, NCS2 family	-	-	Roehe et al., 2016
K03501	16S rRNA (guanine527-N7)-methyltransferase	EC:2.1.1.170	-	Roehe et al., 2016
K03581	Exodeoxyribonuclease V alpha subunit	EC:3.1.11.5	Homologous recombination	Roehe et al., 2016
K03615	Electron transport complex protein RnfC	-	-	Roehe et al., 2016
K03631	DNA repair protein RecN (Recombination protein N)	-	-	Roehe et al., 2016
K03657	DNA helicase II/ATP-dependent DNA helicase PcrA	EC:3.6.4.12	Nucleotide excision repair; Mismatch repair	Roehe et al., 2016
K03694	ATP-dependent Clp protease ATP-binding subunit ClpA	-	-	Roehe et al., 2016
K04112	Benzoyl-CoA reductase subunit C	EC:1.3.7.8	Benzoate degradation; Metabolic pathways; Microbial metabolism in diverse environments; Degradation of aromatic compounds	Li and Guan, 2017
K04130	Muscarinic acetylcholine receptor M2	-	Calcium signaling pathway; cAMP signaling pathway; Neuroactive ligand-receptor interaction; PI3K-Akt signaling pathway; Cholinergic synapse; Regulation of actin cytoskeleton	Li and Guan, 2017
K04517	Prephenate dehydrogenase	EC:1.3.1.12	Phenylalanine, tyrosine and tryptophan biosynthesis; Novobiocin biosynthesis; Metabolic pathways; Biosynthesis of secondary metabolites; Biosynthesis of antibiotics; Biosynthesis of amino acids	Roehe et al., 2016
K04974	Transient receptor potential cation channel subfamily V member 5	-	Parathyroid hormone synthesis, secretion and action; Endocrine and other factor-regulated calcium reabsorption	Shabat et al., 2016
K08483	Phosphotransferase system, enzyme I, PtsI	EC:2.7.3.9	Phosphotransferase system (PTS)	Roehe et al., 2016
K08602	Oligoendopeptidase F	EC:3.4.24.-	-	Roehe et al., 2016
K09811	Cell division transport system permease protein	-	ABC transporters	Roehe et al., 2016
K11752	Diaminohydroxyphosphoribosylaminopyrimidine deaminase/5-amino-6-(5-phosphoribosylamino)uracil reductase	EC:3.5.4.26 or 1.1.1.193	Riboflavin metabolism; Metabolic pathways; Biosynthesis of secondary metabolites; Quorum sensing	Roehe et al., 2016
K13542	Uroporphyrinogen III methyltransferase/synthas	EC:2.1.1.107 or 4.2.1.75	Porphyrin and chlorophyll metabolism; Metabolic pathways; Biosynthesis of secondary metabolites; Microbial metabolism in diverse environments	Roehe et al., 2016

*For simplicity only genes from Shabat et al. (2016) with significant correlation to amino acid metabolism are shown. Genes that lacked function annotation were removed. Genes identified consistently across experiments and therefore representing the most promising marker genes are highlighted in gray*.

**Table 3:** Potential gene biomarkers indicative of methane production from ruminants.

**KEGG number**	**Description**	**E.C number**	**Pathway**	**Published papers**
K00123	Formate dehydrogenase, alpha subunit	EC:1.17.1.9	Glyoxylate and dicarboxylate metabolism; Methane metabolism; Metabolic pathways; Microbial metabolism in diverse environments; Carbon metabolism	Wallace et al., 2015; Roehe et al., 2016; Auffret et al., 2017b
K00125	Formate dehydrogenase, beta subunit	EC:1.17.98.3 or 1.8.98.6	Methane metabolism; Metabolic pathways; Microbial metabolism in diverse environments; Carbon metabolism	Roehe et al., 2016
K00150	Glyceraldehyde-3-phosphate dehydrogenase [NAD(P)]	EC:1.2.1.59	Glycolysis/Gluconeogenesis; Carbon fixation in photosynthetic organisms; Metabolic pathways; Biosynthesis of secondary metabolites; Microbial metabolism in diverse environments; Biosynthesis of antibiotics; Carbon metabolism; Biosynthesis of amino acids	Auffret et al., 2017b
K00169	Pyruvate ferredoxin oxidoreductase, alpha subunit	EC:1.2.7.1	Glycolsis/Gluconeogenesis; Citrate cycle (TCA); Pyruvate metabolism; Nitrotoluene degradation; Propanoate degradation; Butanoate degradation; Methane metabolism; Carbon fixation in proaryotes; Metabolic pathways; Microbial metabolism in diverses environments; Biosynthesis of antibiotics; Carbon metabolism	Roehe et al., 2016; Auffret et al., 2017b
K00170	Pyruvate ferredoxin oxidoreductase, beta subunit	EC:1.2.7.1	As above	Roehe et al., 2016; Auffret et al., 2017b
K00200	Foormylmethanofuran dehydrogenase subunit A	EC:1.2.7.12	Methane metabolism; Metabolic pathways; Microbial metabolism in diverse environments; Biosynthessis of antibiotics; Carbon metabolism	Wallace et al., 2015; Roehe et al., 2016; Auffret et al., 2017b
K00201	Formylmethanofuran dehydrogenase subunit B	EC:1.2.7.12	As above	Wallace et al., 2015; Roehe et al., 2016; Auffret et al., 2017b
K00203	Formylmethanofuran dehydrogenase subunit D	EC:1.2.7.12	As above	Auffret et al., 2017b
K00205	Formylmethanofuran dehydrogenase subunit F	EC:1.2.7.12	As above	Roehe et al., 2016
K00311	Electron-transferring-flavoprotein dehydrogenase	EC:1.5.51	One carbon pool by folate; Carbon fixation pathways in prokaryotes; Metabolic pathways; Microbial metabolism in diverse environments	Kamke et al., 2016
K00323	NAD(P) transhydrogenase	EC:1.6.1.2	Nicotinate and nicotinamide metabolism; Metabolic pathways	Kamke et al., 2016^*^
K00399	Methyl coenzyme M reductase alpha subunit	EC:2.8.4.1	Methane metabolism; Metabolic pathways; Microbial metabolism in diverse environments; Carbon metabolism	Shi et al., 2014; Wallace et al., 2015; Kamke et al., 2016; Roehe et al., 2016; Auffret et al., 2017b
K00400	Methyl coenzyme M reductase system, component A2	EC:2.8.4.1	As above	Roehe et al., 2016; Auffret et al., 2017b
K00401	Methyl coenzyme M reductase system, beta subunit	EC:2.8.4.1	As above	Shi et al., 2014; Wallace et al., 2015
K00402	Methyl coenzyme M reductase system, gamma subunit	EC:2.8.4.1	As above	Shi et al., 2014; Auffret et al., 2017b
K00441	Coenzyme F420 hydrogenase beta subunit	EC:1.12.98.1	Methane metabolism; Metabolic pathways; Microbial metabolism in diverse environments	Roehe et al., 2016
K00539	Oxidoreducatase	EC: 1.97.1.-	-	Kamke et al., 2016
K00577	Tetrahydromethanopterin S-methyltransferase subunit A	EC:2.11.86	Methane metabolism; Metabolic pathways; Microbial metabolism in diverse environments; Biosynthessis of antibiotics; Carbon metabolism	Roehe et al., 2016
K00580	Tetrahydromethanopterin S-methyltransferase subunit D	EC:2.11.86	As above	Roehe et al., 2016; Auffret et al., 2017b
K00581	Tetrahydromethanopterin S-methyltransferase subunit E	EC:2.11.86	As above	Roehe et al., 2016; Auffret et al., 2017b
K00584	Tetrahydromethanopterin S-methyltransferase subunit H	EC:2.11.86	As above	Roehe et al., 2016; Auffret et al., 2017b
K00666	Fatty-acyl-CoA synthase	EC:6.2.1.-	-	Kamke et al., 2016
K00672	Formylmethanofuran-tetrahydromethanopterin N-formyltransferase	EC:2.3.1.101	Methane metabolism; Metabolic pathways; Microbial metabolism in diverse environments; Biosynthessis of antibiotics; Carbon metabolism	Roehe et al., 2016; Auffret et al., 2017b
K00758	Thymidine phosphorylase	EC:2.4.2.4	Pyrimidine metabolism; Drug metabolism–other enzuymes; Metabolic pathways; Bladder cancer	Kamke et al., 2016 (DNA and RNA)
K00814	Alanine transaminase	EC: 2.6.12	Arginie biosynthesis; Alanine, aspartate and glutamate metabolism; Carbon fixation in photosynthetic organisms; Metabolic pathways; Microbial metabolism in diverse environments; Carbon metabolism; 2-oxocarboxylic acid metabolism; Biosynthesis of amino acids	Kamke et al., 2016^*^
K00827	Alanine-glyoxylate transaminase/(R)-3-amino-2-methylpropionate-pyruvate transaminase	EC:2.6.1.44 or EC:2.6.1.40	Metabolic pathways; Biosynthesis of secondary metabolites; Alanine, aspartate and glutamate metabolism; Glycine, serine and threonine metabolism; Cysteine and methionine metabolism; Valine, leucine and isoleucine degradation	Kamke et al., 2016
K00953	FAD synthetase	EC:2.7.7.2	Riboflavin metabolism; Metabolic pathways; Biosynthesis of secondary metabolites	Kamke et al., 2016
K01160	Crossover junction endodeoxyribonuclease RusA	EC:3.1.22.4-	-	Kamke et al., 2016
K01342	Subtilisin	EC:3.4.21.62	Quorum sensing	Kamke et al., 2016
K01479	Formiminoglutamase	EC:3.5.3.8	Histidine metabolism	Shi et al., 2014; Kamke et al., 2016^*^
K01499	Methenyltetrahydromethanopterin cyclohydrolase	EC:3.5.4.27	As above	Roehe et al., 2016; Auffret et al., 2017b
K01631	2-Dehydro-3-deoxyphosphogalactonate aldolase	EC:4.1.2.21	Galactose metabolism; Metabolic pathways	Kamke et al., 2016
K01673	Carbonic anhydrase	EC:4.2.11	Nitrogen metabolism	Auffret et al., 2017b
K01792	Glucose-6-phosphate 1-epimerase	EC:5.1.3.15	Glycolysis/Gluconeogenesis; Metabolic pathways; Biosynthesis of secondary metabolites; Microbial metabolism in diverse environments; Biosynthesis of antibiotics	Kamke et al., 2016
K01846	Methylaspartate mutase	EC:5.4.99.1	C5-Branched dibasic acid metabolism; Purine metabolism	Shi et al., 2014
K01846	Methylaspartate mutase	EC:5.4.99.1	Carbon metabolism; Glyoxylate and dicarboxylate metabolism; C5-Branched dibasic acid metabolism; Metabolic pathways	Kamke et al., 2016
K01912	Phenylacetate-CoA ligase	EC:6.2.1.30	Phenylalanine metabolism; Microbial metabolism in diverse environments; Biofilm formation–Vibrio cholerae	Kamke et al., 2016
K01913	Trans-2-methyl-5-isopropylhexa-2,5-dienoate-CoA ligase	-	-	Kamke et al., 2016
K01959	Pyruvate carboxylase subunit A	EC:6.4.1.1	Citrate cycle (TCA cycle); Pyruvate metabolism; Carbon fixation pathways in prokaryotes; Metabolic pathways; Microbial metabolism in diverse environments; Carbon metabolism; Biosynthesis of amino acids	Auffret et al., 2017b
K02117	V-type H+-transporting ATPase subunit A	EC:3.6.3.14 or EC:3.6.3.15	Oxidative phosphorylation; Metabolic pathways	Wallace et al., 2015; Auffret et al., 2017b
K02118	V-type H+-transporting ATPase subunit B	EC:3.6.3.14 or EC:3.6.3.15	Oxidative phosphorylation; Metabolic pathways	Wallace et al., 2015; Auffret et al., 2017b
K02319	DNA polymerase	EC:2.7.7.7	Pyrimidine metabolism	Shi et al., 2014)
K02319	DNA polymerase I	EC:2.7.7.7	Metabolic pathways; Purine metabolism; Pyrimidine metabolism; DNA replication	Kamke et al., 2016
K02674	Type IV pilus assembly protein PilY1	-	-	Kamke et al., 2016
K02683	DNA primase	EC:2.7.7.-	DNA replication	Kamke et al., 2016
K02856	L-rhamnose-H+ transport protein	-	-	Kamke et al., 2016
K03045	DNA-directed RNA polymerase subunit B″	EC:2.7.7.6	Purine metabolism; Pyrimidine metabolism; Metabolic pathways; RNA polymerase	Kamke et al., 2016^*^
K03053	DNA-directed RNA polymerase subunit H	EC:2.7.7.6	Purine metabolism; Pyrimidine metabolism; Metabolic pathways; RNA polymerase	Kamke et al., 2016^*^
K03124	Transcription initiation factor TFIIB	-	Basal transcription factors; Epstein-Barr virus infection; Viral carcinogenesis	Kamke et al., 2016^*^
K03167	DNA topoisomerase VI subunit B	EC:5.99.1.3	-	Kamke et al., 2016^*^
K03219	Type III secretion protein SctC	-	Bacterial secretion system	Kamke et al., 2016
K03222	Type III secretion protein SctJ	-	as above	Kamke et al., 2016
K03223	Type III secretion protein SctL	-	as above	Kamke et al., 2016
K03224	ATP synthase in type III secretion protein SctN	EC:3.6.3.14	as above	Kamke et al., 2016
K03226	Type III secretion protein SctR	-	as above	Kamke et al., 2016
K03227	Type III secretion protein SctS	-	as above	Kamke et al., 2016
K03228	Type III secretion protein SctT	-	as above	Kamke et al., 2016
K03229	Type III secretion protein SctU	-	as above	Kamke et al., 2016
K03230	Type III secretion protein SctV	-	as above	Kamke et al., 2016
K03579	ATP-dependent helicase HrpB	EC:3.6.4.13	-	Kamke et al., 2016
K04058	Type III secretion protein SctW	-	Bacterial secretion system	Kamke et al., 2016
K04795	Fibrillarin-like pre-rRNA processing protein	-	-	Kamke et al., 2016^*^
K04801	Replication factor C small subunit	-	-	Kamke et al., 2016^*^
K04835	Methylaspartate ammonia-lyase	EC:4.3.1.2	C5-Branched dibasic acid metabolism	Shi et al., 2014
K04857	Voltage-dependent calcium channel L type alpha-1S	-	MAPK signaling pathway; Calcium signaling pathway; cGMP-PKG signaling pathway; cAMP signaling pathway; Cardiac muscle contraction; Adrenergic signaling in cardiomyocytes; Vascular smooth muscle contraction; Retrograde endocannabinoid signaling; Cholinergic synapse; Serotonergic synapse; GABAergic synapse; Insulin secretion; GnRH signaling pathway; Oxytocin signaling pathway; Renin secretion; Aldosterone synthesis and secretion; Cortisol synthesis and secretion; Cushing's syndrome; Alzheimer's disease; Hypertrophic cardiomyopathy (HCM); Arrhythmogenic right ventricular cardiomyopathy (ARVC); Dilated cardiomyopathy (DCM)	Kamke et al., 2016^*^
K04874	Potassium voltage-gated channel Shaker-related subfamily A member 1	-	-	Kamke et al., 2016
K05830	Acetylornithine/LysW-gamma-L-lysine aminotransferase	EC:2.6.1.11	Arginine biosynthesis; Lysine biosynthesis; Metabolic pathways; Biosynthesis of secondary metabolites; Biosynthesis of antibiotics; 2-Oxocarboxylic acid metabolism; Biosynthesis of amino acids	Kamke et al., 2016
K06863	(Beta)-D-ribofuranosyl 5′-monophosphate synthetase	EC:6.3.4.-	Purine metabolism	Shi et al., 2014
K06863	5-Formaminoimidazole-4-carboxamide-1-(beta)-D-ribofuranosyl 5′-monophosphate synthetase	EC:6.3.4.23	Purine metabolism; Metabolic pathways; Biosynthesis of secondary metabolites; Biosynthesis of antibiotics	Kamke et al., 2016^*^
K06907	Phage tail sheath protein FI	-	-	Kamke et al., 2016^*^
K06927	Diphthine-ammonia ligase	EC:6.3.1.14	-	Kamke et al., 2016^*^
K06937	7,8-dihydro-6-hydroxymethylpterin dimethyltransferase	EC:2.1.1.-	-	Auffret et al., 2017b
K07249	Retinal dehydrogenase	EC:1.2.1.36	Retinol metabolism; Metabolic pathways	Kamke et al., 2016
K07283	Putative salt-induced outer membrane protein	-	-	Kamke et al., 2016
K07318	Adenine-specific DNA-methyltransferase	EC:2.1.1.72	-	Kamke et al., 2016^*^
K07463	Archaea-specific RecJ-like exonuclease	-	-	Kamke et al., 2016^*^
K07558	tRNA nucleotidyltransferase (CCA-adding enzyme)	EC:2.7.7.72	-	Kamke et al., 2016^*^
K07569	RNA-binding protein	-	-	Kamke et al., 2016^*^
K07732	Riboflavin kinase, archaea type [EC:2.7.1.161]	EC:2.7.1.161	Riboflavin metabolism; Metabolic pathways	Kamke et al., 2016^*^
K07796	Cu(I)/Ag(I) efflux system outer membrane protein CusC/SilC	-	-	Kamke et al., 2016
K08605	Coccolysin [EC:3.4.24.30]	EC:3.4.24.30	Quorum sensing	Kamke et al., 2016
K08635	Membrane metallo-endopeptidase-like 1	-	-	Kamke et al., 2016
K08636	Phosphate-regulating neutral endopeptidase	EC:3.4.24.-	-	Kamke et al., 2016
K08641	D-alanyl-D-alanine dipeptidase [EC:3.4.13.22]	EC:3.4.13.22	Vancomycin resistance; Two-component system	Kamke et al., 2016
K09482	Glutamyl-tRNA (Gln) amidotransferase subunit D	EC:6.3.5.7	Aminoacyl-tRNA biosynthesis	Shi et al., 2014
K09482	Glutamyl-tRNA(Gln) amidotransferase subunit D	EC:6.3.5.7	Aminoacyl-tRNA biosynthesis; Metabolic pathways	Kamke et al., 2016^*^
K09610	Endothelin-converting enzyme-like 1	EC:3.4.24.-	-	Kamke et al., 2016
K10060	C-type lectin domain family 4 member F	-	-	Kamke et al., 2016
K10639	E3 ubiquitin-protein ligase CCNP1IP1	EC:6.3.2.19	-	Kamke et al., 2016
K10725	Archaeal cell division control protein 6	-	-	Kamke et al., 2016^*^
K10896	Fanconi anemia group M protein	-	Fanconi anemia pathway	Kamke et al., 2016^*^
K11382	MFS transporter, OPA family, phosphoglycerate transporter protein	-	Two-component system	Kamke et al., 2016
K11404	Histone deacetylase 3	EC:3.5.1.98	Thyroid hormone signaling pathway; Alcoholism; Human papillomavirus infection; Viral carcinogenesis	Kamke et al., 2016
K11900	Type VI secretion system protein ImpC	-	Biofilm formation–Pseudomonas aeruginosa	Kamke et al., 2016
K12204	Defect in organelle trafficking protein DotC	-	-	Kamke et al., 2016
K12206	Intracellular multiplication protein IcmB	-	-	Kamke et al., 2016
K12217	Intracellular multiplication protein IcmO	-	-	Kamke et al., 2016
K12221	Intracellular multiplication protein IcmS	-	-	Kamke et al., 2016
K12434	Polyketide synthase 7	-	-	Kamke et al., 2016
K12448	UDP-arabinose 4-epimerase	EC:5.1.3.5	Amino sugar and nucleotide sugar metabolism; Metabolic pathways	Kamke et al., 2016
K12739	Peptidyl-prolyl cis-trans isomerase-like 6	EC:5.2.1.8	-	Kamke et al., 2016^*^
K13085	Phosphatidylinositol-4,5-bisphosphate 4-phosphatase	EC:3.1.3.78	Bacterial invasion of epithelial cells; Shigellosis; Salmonella infection	Kamke et al., 2016
K13600	Chlorophyllide a oxygenase	EC:1.14.13.122	Porphyrin and chlorophyll metabolism; Metabolic pathways; Biosynthesis of secondary metabolites	Kamke et al., 2016
K13670	Putative glycosyltransferase	EC:2.4.-.-	-	Kamke et al., 2016
K13812	Bifunctional enzyme Fae/Hps	EC:4.2.1.147 or 4.1.2.43	Pentose phosphate pathway; Methane metabolism; Metabolic pathways; Microbial metabolism in diverse environments; Carbon metabolism; Biosynthessis of amino acids	Roehe et al., 2016; Auffret et al., 2017b
K13886	Coronin-1B	-	-	
K13893	Microcin C transport system substrate-binding protein	-	ABC transporters	Kamke et al., 2016
K14123	Energy-converting hydrogenase B N	-	-	Roehe et al., 2016
K14128	F420-non-reducing hydrogenase subunit G	EC:1.12.99.- or EC:1.8.98.5	Methane metabolism; Metabolic pathways; Microbial metabolism in diverse environments; Carbon metabolism	Roehe et al., 2016; Auffret et al., 2017b
K14275	D-xylonate dehydratase	EC:4.2.1.82	Pentose and glucuronate interconversions	Kamke et al., 2016
K14324	Histone deacetylase complex subunit SAP18	-	RNA transport; mRNA surveillance pathway	Kamke et al., 2016
K14333	2,3-dihydroxybenzoate decarboxylase	EC:4.1.1.46	Benzoate degradation; Aminobenzoate degradation	Shi et al., 2014
K14333	2,3-dihydroxybenzoate decarboxylase	EC:4.1.1.46	Benzoate degradation; Aminobenzoate degradation; Microbial metabolism in diverse environments	Kamke et al., 2016
K14414	Transcriptional regulatory protein RtcR	-	-	Kamke et al., 2016
K14426	Solute carrier family 12 (sodium/chloride transporter), member 3	-	-	Kamke et al., 2016
K14429	Solute carrier family 12 (potassium/chloride transporters), member 9	-	-	Kamke et al., 2016
K14995	Solute carrier family 38 (sodium-coupled neutral amino acid transporter), member 9	-	mTOR signaling pathway	Kamke et al., 2016^*^

*Data are from metagenomics sequences unless marked with an asterisk*.

* *Indicates metatranscriptomic datas. Genes that lacked functional annotation were removed. Genes identified consistently across experiments and therefore representing the most promising marker genes are highlighted in gray*.

## Prospects for enhancing rumen microbiome understanding and animal phenotype predictions via mathematical modeling

Mathematical models can be used to integrate our understanding of feed, intake, digestion and passage rates on the resulting energy available to the microbiome and ultimately the host. The development of rumen models has been deployed mainly *via* the consolidation of four model structures (Molly, Karoline, Cornell, and Dijkstra models) that have been improved over the years to enhance the understanding of rumen function (Mills et al., 2014; Huhtanen et al., 2015; Van Amburgh et al., 2015; Gregorini et al., 2016). These models represent relevant aspects that determine the nutritional and emission responses for a given diet but do not attempt to provide a detailed description of the microbiota or its function (Ellis et al., 2008). This gap between the available omics data of the rumen microbiome and the models needs to be bridged to improve our understanding of rumen function (Bannink et al., 2016; Muñoz-Tamayo et al., 2016). To make these model applications possible, rumen modeling should embrace the framework of genome-scale metabolic models (GEMs). The basis of a GEM is the stoichiometry matrix that links metabolites and biochemical reactions that the microbe is able to perform as a result of its genetic potential. The stoichiometry matrix is organism-specific and results from a genome-scale network reconstruction obtained by a protocol that includes functional genome annotation, curation of a draft reconstruction of metabolic reactions and finally translation of the reconstructed network into a computational model (GEM). The full process capitalizes on high-throughput network-wide and bibliomic data (Feist et al., 2009), and on dedicated software (Henry et al., 2010; Aite et al., 2018). The construction of a rumen microbiome GEM will need to address central questions that remain to be elucidated due to the early stage of microbial community modeling (Zengler and Palsson, 2012). One of these key questions is how microbial species, their metabolic networks, and interspecies interactions should be represented (Biggs et al., 2015). Once this question is elucidated, a plethora of constraint-based reconstruction and analysis (COBRA) methods can be deployed to investigate genotype–phenotype relationships (Lewis et al., 2012).

The COBRA methods rely on the principle that microbial metabolism is bound by constraints that include thermodynamics, substrate and enzyme availability. These methods mainly operate under steady-state. The most popular COBRA method is flux balance analysis (FBA; Varma et al., 1993; Varma and Palsson, 1994), which looks at finding the network reaction fluxes that optimize a regulatory condition (e.g., microbial growth). Overall, COBRA approaches provide rational tools for metabolic engineering. The number of applications is broad and includes the development of tools for (i) studying interactions among different microbial groups, i.e., protozoa, fungi, archaea, bacteria and viruses or bacteriophages, (ii) developing selective cultivation strategies for as yet uncultured rumen microbes (Pope et al., 2011), (iii) designing methane mitigation strategies by exploiting the metabolic networks of genome-sequenced rumen archaea (Leahy et al., 2010; Pope et al., 2011), and (iv) developing prediction tools that exploit microbiome biomarkers for fiber hydrolysis (Dai et al., 2015; Comtet-Marre et al., 2017, 2018) and methane production (Popova et al., 2013; Shi et al., 2014; Auffret et al., 2017b).

Clearly rumen GEMs must be further integrated into whole rumen digestion models to provide a system-level picture of the dynamic interplay between the diet, the animal host and the rumen microbiota. Central to this task and for the development of novel strategies to enhance ruminant production and reduce environmental impact is the need for data sharing and collaboration. The co-authors of this paper are all members of the Global Research Alliance's Rumen Microbial Genomics Network, which is set up to allow global collaborations and data sharing for this very purpose. This integration task is far from trivial due to multiple time scales, among other aspects such as parameter identifiability (Muñoz-Tamayo et al., 2018). Moreover, since COBRA approaches mainly operates at steady-state, dynamic frameworks (Mahadevan et al., 2002; Baroukh et al., 2014) will need to be adapted to account for the dynamic fluctuations within the rumen environment. A great challenge is to deploy different model structures, capitalizing on “omics” data, and responding to different goals varying from supporting livestock management within a precision farming context to guiding microbial programming strategies.

## Technological advances to further our understanding of the rumen microbiome

### Genomics/culturomics

Large culture collections are incredibly powerful as the organisms in the collection can be studied both *in vitro* and *in vivo*. However, they may also be limited to, or biased toward, strains that are easy to culture, highly abundant organisms and organisms which are of specific interest to research. Since the seminal work of Robert Hungate published in his book “The rumen and its microbes” in 1966 (Hungate, 1966), technologies have rapidly advanced. The foundational work of Robert Hungate formed the backbone of the Hungate1000 project (Seshadri et al., 2018) led by AgResearch, New Zealand and formed a major project within the Global Research Alliance's Rumen Microbial Genomics Network. It's aims were to sequence 1,000 cultured rumen microbial genomes to aid our understanding of the rumen microbiome. The Hungate1000 project recently finished having sequenced 420 representatives of rumen microbes (mainly bacteria), and thus providing a major tool for the community (Seshadri et al., 2018).

However, many of the rumen bacteria remain uncultured and uncharacterized, with genomic information on the rumen eukaroytes being especially sparse in the Hungate1000 genomes as a result of the challenges of sequencing the A-T rich genomes of these microbes. Our ability to culture rumen bacteria has improved in recent years through the development of culture media (Kenters et al., 2011). A recent study by Poelaert et al. (2018) showed that reducing agents were not required to culture all rumen bacteria and that when removed they resulted in higher microbial diversity. Techniques such as dilution to extinction have also improved our ability to culture bacteria in many ecosystems, including the rumen (Kenters et al., 2011). Another method, which has had success for culturing marine bacteria, is the microdroplet encapsulation technique (Zengler et al., 2002). This involves using a version of the natural environment by using a dilution to extinction technique, followed by encapsulation in a gel and suspending microdroplets in a column. The medium from the environment in which the bacterium was isolated can then be flowed through to provide nutrients for growth (Stewart, 2012). Indeed, there are many technologies emerging that should be investigated for their ability to culture the as yet unculturable rumen bacteria.

### Metataxonomy and inference of function

The onset of next generation sequencing resulted in an explosion in publications exploring the metataxonomy of the rumen microbiome under differing parameters. Although these studies are of great value, interpretation of the data generated across different publications remains a challenge. Differences among studies exist with respect to DNA extraction, primers and cycling parameters, as well as downstream computational analysis, resulting in conflicting data (Yu and Morrisson, 2004a,b; Edwards et al., 2007; Huws et al., 2007; Kim et al., 2011; Ishaq and Wright, 2014; Vaidya et al., 2018). With respect to DNA extraction Yu and Morrisson (2004a) evaluated three extraction techniques (a modified phenol-free bead-beating method (referred to as repeated bead beating plus column (RBB + C) method, FastDNA SPIN Kit (MP Biomedicals, California) and the QIAamp DNA Stool Mini Kit (Qiagen, Germany). They concluded that the RBC + C method yielded more DNA and that bead beating was crucial. Vaidya et al. (2018) further tested 4 DNA extraction methods (Repeated bead beating (RBB) developed by Yu and Morrisson (2004a), phenol dependent bead beating (PBB), Fast SPIN DNA kit for soil (MP Biomedicals, California), and the PQIAmini) using both rumen fluid and fibrous rumen samples. The authors concluded that each method was effective but gave different results, for example PBB extracted DNA extracted resulted in higher abundances of Ruminococcaceae compared with abundances obtained using the FDSS method, whereas abundances of Fibrobacteraceae was lower compared with the RBB method. They conclude that each method has advantages and disadvantages which need to be considered based on sample type, but bead beating is critical. Further downstream many authors have investigated the importance of primer choice for metataxonomic investigations of the rumen microbiome (Yu and Morrisson, 2004b; Edwards et al., 2007; Huws et al., 2007; Kim et al., 2011; Ishaq and Wright, 2014). Yu and Morrisson (2004b) investigated primer choice in terms of diversity observed on denaturing gradient gel electrophoresis (DGGE) gels and concluded that primers targeting the V3 region were the best. Huws et al. (2007) and Edwards et al. (2007), however showed that the V3 primers were non-specific to bacteria and could amplify plant chloroplastic DNA as well as archaeal 16S rDNA and protozoal 18S rDNA sequences. The amplification of plant chloroplast sequences is a substantial issue for samples taken from animals fed fresh forage (these are often assigned taxonomically as cyanobacteria), with an abundance of intact chloroplast DNA being present in the rumen, and often masks microbial sequences (Edwards et al., 2007). This is the case when investigated using DGGE and NGS based sequencing (Huws, personal communication). Nonetheless, if animals are not fed fresh forage and primers which are more broad are required to cover a greater proportion of the microbes as a whole is required then the V3 region is perhaps a justifiable choice. However, Huws et al. (2007) concluded that for specific bacterial 16S rRNA V6-V8 primers were more appropriate. Edwards et al. (2007) also developed a primer pair based on the V6-V8 region which reduce the amplification of chloroplastic DNA. Nonetheless, the amplicon size obtained using the Edwards et al. (2017) primers are often too large for effective sequencing, therefore the reverse primer has been changed to enable avoidance of chloroplast identification and production of a smaller amplicon for NGS sequencing (Belanche et al., 2017). The annealing temperatures and number of cycles used for PCR are also clearly going to bias results somewhat. Nonetheless, using a basic set of standardized protocols may not be possible, due to the differing hypotheses and the complex nature of the ecosystem (i.e., host animal), however ensuring data accuracy by using internal standards represents one approach to ensure that comparisons among datasets are valid. Pollock et al. (2018) attempted to describe the guidelines and consensus best practices for metataxonomic studies and concluded that bead beating is critical for DNA extraction as is the use of internal standards for metataxonomic studies amongst other recommendations. Also the construction of rumen microbiome databases to aid accurate taxonomical assignment, such as RIM-DB (for methanogens; Seedorf et al., 2014), the ureC database (ureolytic bacteria; Jin et al., 2017), and AF-RefSeq (anaerobic fungi; Paul et al., 2018) drastically improve our ability to monitor rumen microbial diversity.

Irrespective, metataxonomic rDNA data have provided insights into the composition of the rumen microbiome under differing parameters, but these techniques are limited in terms of providing insight into microbial function. Nonetheless, due to their low cost, these techniques are the most published and will continue to be important in microbiome research for the near future. Software to predict microbial function from metataxonomic data, such as PICRUSt (Langille et al., 2013), has been applied to many different ecosystems, including the rumen. Although this approach saves on the cost associated with more thorough and accurate shotgun metagenomic analysis, it has limitations in accurately represent microbiome function (Wilkinson et al., 2018). The accuracy of PICRUSt prediction, originally intended for human microbiota data, has recently been tested for the rumen microbiome using datasets with 16S rDNA data and accompanying metagenomics or metatranscriptomic datasets (Wilkinson et al., 2018). The data shows poor correlation of predicted function with the actual function seen within the metagenomics/metatranscriptomic datasets (Wilkinson et al., 2018). Wilkinson et al. (2018), developed CowPI an improved 16S rDNA inference platform for the rumen which is based on PICRUSt but uses the Hungate1000 genomes as the searchable genomes (http://www.cowpi.org/). Other platforms allowing inference of function from 16S rDNA data have also been developed, such as Tax4Fun (Aßhauer et al., 2015) and PanFP (Jun et al., 2015), and have been proposed to provide more accurate functional annotations than PICRUSt (Koo et al., 2017). However, the ability of these programs to predict the function of the rumen microbiome has not been investigated. Regardless, inferring metabolic function from phylogenetic data allows the scientific community to obtain retrospective value from these datasets in order to understand the rumen microbiome in light of global agricultural challenges.

### Metagenomics

The benefits of metagenomics include the ability to assemble whole- and fragmented-genomes, predict genes, map enzymes and pathways, discover new enzymes and pathways, and quantify the abundance of functional genomic elements across and between samples. Shotgun metagenomics was first applied to the rumen in order to discover novel biomass degrading enzymes from switchgrass-associated microbes (Hess et al., 2011). Subsequently, metagenomics has been used to study many aspects of rumen microbiology, including methane emissions in cattle (Wallace et al., 2015) and sheep (Shi et al., 2014), biomarkers to predict ruminal methanogenesis (Auffret et al., 2017b), the effect of feed-conversion-ratio, and breed and host genetics on the composition of the rumen microbiome (Roehe et al., 2016), nutrient acquisition (Mayorga et al., 2016; Rubino et al., 2017), and effects of diet (Auffret et al., 2017a,b), and investigate impact of feed additives (Thomas et al., 2017) on the abundance of antimicrobial-resistance genes. The rumen also remains a source of valuable bioactives for the biotechnology industry, and metagenomics is a key tool for such bioprospecting (Oyama et al., 2017; Roumpeka et al., 2017). More recently, metagenomic sequences have also resulted in an enhanced understanding of niche specialization within rumen bacteria (Rubino et al., 2017). Rubino et al. (2017) showed that, within metagenome sequences from 14 silage-fed cows, that the genus *Prevotella* possessed higher levels of glycosyl hydrolase (GH) isoforms relating specifically to the degradation of hemicellulose, whilst *Clostridium* contained higher levels of GH isoforms for enzymes specifically involved in cellulose degradation. Their data suggests that isoform diversity maintains selective advantage and niche specialization within these genera.

### Binning genomes from metagenomes

Another major advancement in understanding the capacity of rumen bacteria has been our increased ability to bin genomes from metagenomes. Assembly binning refers to the construction of complete or near complete microbial genomes directly from metagenomic sequencing data, and was first achieved by Tyson et al. (2004) from an acidophilic biofilm. Hess et al. (2011) were the first to apply this to ruminants, assembling 15 draft microbial genomes from the switchgrass associated microbiome of cattle. Subsequently, Svartstrom et al. (2017) assembled 99 microbial genomes from the moose rumen, Stewart et al. (2018) assembled 913 microbial genomes from the rumen of cattle, and Parks et al. (2017) assembled over 8,000 novel microbial genomes from 1,500 public datasets, some of which originated from the rumen. Traditional metagenomic binning takes an *in silico* approach whereby metagenomic assembled contigs are clustered by base-composition and abundance across multiple datasets—the hypothesis being that contigs from the same organism will follow a very similar abundance profile across multiple samples, and will have a roughly similar base composition. The success of such binning procedures is validated by investigating the number of single-copy core-genome genes within each bin, as implemented by software such as CheckM (Parks et al., 2015). More recently, physical methods of metagenomic binning such as the use of Hi-C have been published (Beitel et al., 2014). In Hi-C experiments, parts of the chromosome that are in contact with one another inside the cell are cross-linked using formaldehyde; cells are then lysed, the DNA is fragmented using a restriction enzyme, followed by random ligation, amplification and sequencing. Each pair of paired-end reads therefore comes from two separate fragments of the same original chromosome, and that information can be used to collate assembled contigs into genomes. Hi-C binning has been used effectively on human feces (Press et al., 2017) as well as in ruminants (Stewart et al., 2018).

### Metatranscriptomics

Metatranscriptomics involves the profiling of community-wide expressed genes (mRNA), and is often termed RNA-seq. Whilst metagenomics allows us to evaluate diversity and the potential functional capacity of a microbiome, metatranscriptomes provide insight into the actual function of microbiomes via gene expression. Due to the abundance of rRNA, metatranscriptomics requires either very deep sequencing to obtain sufficient mRNA sequences coupled with computational binning of the rRNA genes (these can also be useful for metataxonomics) or use of kits to deplete rRNA pre-sequencing. Deep sequencing is of course expensive and the kits used to remove rRNA for metranscriptomics of the rumen microbiome, have varying degrees of success (Huws personal communication). These kits are also bespoke for the removal of bacterial or eukaryotic rRNA, and thus for the complex rumen microbiome, a variety of kits are required to remove prokaryotic and eukaryotic rRNA. This is both costly and laborious, with the time required likely resulting in partial RNA degradation, which will ultimately bias down-stream analysis. Nonetheless Comtet-Marre et al. (2017) developed a bespoke rRNA kit which was effective in removing rumen microbial rRNA, providing a potential solution for future experiments.

Despite these developments, it has also been shown that the correlation between mRNA and protein levels can be weak and variable, possibly due to post-transcriptional modifications (Greenbaum et al., 2003; Csárdi et al., 2015). Ribosome profiling (riboseq) has been developed as a direct method to quantify and characterize translation (Ingolia et al., 2009). Riboseq takes advantage of the fact that during translation, the ribosome protects around 30 nucleotides of the mRNA from nuclease activity. High-throughput sequencing of these ribosome protected fragments offers a precise record of the number and location of the ribosomes at the time translation ceases. Mapping the position of the ribosome-protected fragments is indicative of the translated regions within the transcriptome. Nonetheless, whilst the use of this technique on pure cultures has been effective, the development of the technique (MetaRibo-Seq) at a metatranscriptomic level is in its infancy and still requires validation for the rumen micobiome.

### Metaproteomics

Metaproteomics falls between the established DNA and RNA sequencing and metabolomics procedures as an approach to characterize the functional activity of the microbial community. While still an emerging technology, the concept was introduced by Wilmes and Bond (2004), who used 2D PAGE methods to separate and identify proteins from a complex sample extracted from waste water treatment. As a concept, it has some theoretical advantages over RNA sequencing methods in that the half-life of proteins can be significantly longer than RNA transcripts. Therefore, if the data represent a “snapshot” of microbial activity at a single time point, then identification of the proteins arguably will provide a more accurate picture than sequencing mRNA. Moreover, as individual proteins can be identified by their amino acid sequence, function can still be linked to taxa using protein sequence alignment tools e.g., UniPept (Mesuere et al., 2018).

The 2D PAGE methodology involves using a pH gradient firstly to separate proteins in one dimension based on their isoelectric point. The proteins are then subject to gel electrophoresis to separate them by size. This results in a spot pattern representing the metaproteome. Individual spots can then be excised, digested and the resulting peptides identified using mass spectrometry. This method has been used to identify proteins in waste water samples (Abram et al., 2009) soils, sediments (Benndorf et al., 2009; Chourey et al., 2010), the rhizosphere (Wu et al., 2011) and human feces (Klaassens et al., 2007). However, when applied to rumen digesta samples this approach revealed a major shortcoming (Snelling and Wallace, 2017), as the rumen contains high levels of plant secondary compounds, such as tannins and other phenolics that complex with the proteins, and interfere with protein extraction and purification (Snelling and Wallace, 2017). Snelling and Wallace (2017) reported that repeated wash steps and microfiltration were not effective in removing the contaminants, which prevent accurate protein quantitation and obscure spot patterns in gels. Humic acid is also highly abundant in soil and feces, which cause similar implications for recovery for good metaproteomic data in these systems. One possible solution to this problem is to use acid precipitation to separate peptides (Qian and Hettich, 2017). The authors concluded that sample quality was a key factor, with best results obtained from fresh digesta or samples with high microbial protein content relative to contaminants. Despite these limitations, Snelling and Wallace (2017) did identify proteins directly associated with the functional activity of the rumen microbial community from 2D PAGE spots. Abundant structural proteins were identified including actin, alpha and beta tubulin, and axonemal isoforms dynein light chain, which are all involved in the locomotion of ciliates. Among prokaryotic proteins were enzymes from the Phyla Firmicutes and Bacteroidetes involved in central metabolism. Archaeal proteins were also found in surprisingly high abundance considering the relatively small proportion of the microbial community that this group occupies in the rumen. This may be a reflection of the persistence of archaeal proteins after the original transcripts are degraded. These archaeal proteins, were identified as key enzymes involved in the synthesis of methane. This finding was particularly relevant to the efforts to understand the mechanisms behind methane production.

As a consequence of these technical challenges, very few studies have attempted to characterize the rumen microbial community using metaproteomics. However, in recent years, the development of next generation mass spectrometers and accompanying software have provided the means to identify proteins *en masse* in an approach to analogous to shotgun DNA sequencing. Using this technique Deusch and Seifert (2015) described over 2,000 proteins associated with the rumen microbial community. Research recently conducted by Hart et al. (2018) explored the potential to develop a metaproteomic approach to analyse the rumen that allowed comparison of data with meta-transcriptomic information. Although in its infancy, the meta-proteomic methodology did allow for the identification of members of the protein families that were associated with the transcriptome of the rumen microbiome (Hart and Kingston-Smith, Personal Communication). The development of software for meta-proteome analysis such as Meta-proteome Analyzer (Muth et al., 2015) can also aid in the analysis and interpretation of meta-protein data. As it stands the method shows great potential and is a complement to other omics technologies to determine the functionality of the rumen microbiome.

### Metabolomics

Like the field of proteomics, the study of metabolomics within the rumen is also in its infancy. Metabolomics can be defined as the comprehensive (qualitative and quantitative) analysis of metabolites by gathering as much metabolic information as possible from an organism or biological system (Yi et al., 2016). Metabolomics focuses mainly on low molecular mass molecules (<1,000 Da), which can be related to the functional status of the organism (Bundy et al., 2009). The main challenges for analyzing these metabolites include their chemical complexity and heterogeneity. Sample preparation for metabolomics can be as simple as a liquid-liquid extraction procedure, but using assertive methods is key to ensure effective metabolite extraction (Patejko et al., 2017). Various analytical instruments can be used for metabolomics, differing mainly on sensitivity and coverage. To date, the majority of rumen metabolome studies have used liquid chromatography-mass spectrometry (LC-MS), gas chromatography-mass spectrometry (GC-MS) and nuclear magnetic resonance (NMR), with the latter the most used due to reliability and absolute quantification (Goldansaz et al., 2017). There is growing interest for LC-MS due to recent advances in instrument sensitivity, high processing capacity, data analysis and the development of data repositories where the community can curate large data sets have brought interest to tandem mass spectrometry (MS). Furthermore, with the introduction of the online tool Global Natural Products Social Molecular Networking, a crowd-sourced knowledge repository and analysis infrastructure, whereby MS/MS spectra can be clustered based on spectral similarity data, and greatly improving data interpretation (Wang M. et al., 2016).

Usually, two approaches can be used for metabolic investigations: targeted and untargeted analysis. The targeted analysis focuses on examination of a group of known metabolites, usually for hypothesis-driven studies (Patti et al., 2012), whereas untargeted analysis evaluates large numbers of compounds. One of the main advantages of using an untargeted approach is the prospection of novel compounds and metabolic pathways (Patti et al., 2012). To date, most studies regarding the rumen metabolome used the targeted approach and had an interest in the effect of diets on the rumen metabolome (Ametaj et al., 2010; Saleem et al., 2012, 2013; Zhao et al., 2014; Zhang et al., 2015; Mao et al., 2016, 2017; Zhang R. et al., 2017; Do Prado et al., 2018; O'callaghan et al., 2018). Using a combination of several metabolomics platforms, the pioneer study by Saleem et al. (2013) demonstrated that the rumen metabolome is not as simple as previously anticipated. Indeed, 246 compounds were reported as part of the rumen metabolome, including: phospholipids, inorganic ions, gases, amino acids, dicarboxylic acids, fatty acids, volatile fatty acids, glycerides, carbohydrate and cholesterol esters (Saleem et al., 2013). As part of this study Saleem et al. (2013) also set up a searchable rumen metabolome database to improve metabolite assignments in the rumen (www.rumendb.ca). Recent studies have mainly explored how rumen metabolites are affected by different levels of roughages and concentrate. For example, there is alteration to organic acids, amino acids, amines, sugars and nucleosides/nucleotides when cows were fed diets low on concentrate (40%, DM basis) compared to high concentrate (70%, DM basis; Zhang R. et al., 2017). In another study using dairy cows fed diets varying in roughage to concentrate ratio (80:20, 60:40, 40:60, and 20:80) ruminal amino acids, lipids, organic acids, and carbohydrates were affected (Zhang J. et al., 2017).

Metabolites of microbial origin are also precursors of ruminant products (e.g., meat and milk), which might suggest biochemical insights into the role played by rumen-diet interactions (Saleem et al., 2013; Sun et al., 2015). Also, metabolites such as phosphatidylcholine have been suggested as biomarkers for protozoa abundance (Saleem et al., 2012). Finally, the rumen fluid metabolome can be used to identify potential differences in rumen function. Recently, Artegoitia et al. (2017) reported 33 metabolites differing in cattle having high or low average daily gain. Kingston-Smith et al. (2013), using Fourier Transform Infra-Red (FT-IR) spectroscopy-based metabolite profiling, also showed that it was possible to discriminate differences in the rumen plant-microbe interactome when three different cultivars of perennial ryegrass were used as substrate. This suggests that FT-IR could be used as an approach to improve forages for livestock production (Kingston-Smith et al., 2013). However, there are few studies to date combining both sequencing and metabolomics to provide a more comprehensive analysis of the rumen system that would contribute information regarding metabolic expression of the genetic potential of the microbiota (Mao et al., 2016; Zhang J. et al., 2017; O'callaghan et al., 2018).

The rumen environment is composed of a myriad of molecules of microbial, plant and animal origins. Currently, studies have focused on characterizing how the ruminal environment is affected by diet. There is an opportunity to extend this to explore microbial interactions and how ruminal microbes cope with stressors. Concentrating on exploring the ecological foundations of the rumen microbiota might deliver an improved comprehension of the rumen, novel compounds and unexplored pathways.

### Data integration for enhanced understanding of the rumen microbiome

Each of the previously described technological approaches provide novel and fascinating insights into aspects of the structure, function and/or activity of the rumen microbiome. However, if these different types of information were to be integrated into a model that describes microbial activity at all levels recorded, our understanding would be further enhanced. At the simplest level we can use the central dogma of molecular biology that describes the manner in which genetic information is transcribed and translated to proteins (DNA = > RNA = > Protein) to allow the “functional potential” in whole sequenced genomes from cultured rumen microbes, metagenomically assembled genomes (MAGs) or *de novo* assembled metagenomes to be linked to the actively transcribed genes captured using whole transcriptomes from cultured microbes, or meta-transcriptomic data from rumen samples. This level of data integration has provided some novel insights missed by either approach alone, such as by Shi et al. (2014) who demonstrated that differences in methane production were not due to differences in the abundances of the genes responsible, but rather a result of their differential expression. It would not have been possible to make this distinction without integration of both types of data. Similarly, the comparison of proteomic data to transcriptomic or genomic data from the same samples can allow better protein identification (using tools such as MASCOT) and provide results that are more relevant to the rumen environment (Hart et al., 2018).

This, however, is only scratching the surface of what is possible. For example, online databases, such as the Kyoto Encyclopedia of Genes and Genomes (KEGG; Kanehisa et al., 2017) and BioCyc (Caspi et al., 2016), which provide collections of characterized metabolic pathways and software tools for exploring them, have been used to provide a better understanding of rumen microbial activity in a variety of contexts (Hess et al., 2011; Shi et al., 2014; Rubino et al., 2017; Seshadri et al., 2018). However, many of these databases are targeted toward human pathogens or model aerobic organisms, such as *Escherichia coli*, and do not necessarily reflect the functions important to the facultative anaerobic lifestyle of rumen microbes. With nearly 500 genomes from rumen organisms currently available, an opportunity exists to generate rumen-specific databases of known and predicted metabolic functions, similar to INTERMINE (Kalderimis et al., 2014) although this will likely require a parallel concerted effort to generate better phenotypic information for these cultured organisms *in vitro* and *in vivo*.

The ultimate goal is to be able to combine information from all types of “omics” data, and provide a true “systems” overview of the rumen microbiome. Progress toward achieving this continues for a few other model microbiomes, where the goal has been to identify interactions in order to interpret biological data and ultimately model *in silico* how microbial communities behave (Bittner et al., 2010; Faust and Raes, 2012). This is generally accomplished through three tasks: firstly by identifying the “scaffold” of interactions between organisms (Faust and Raes, 2012; Friedman and Alm, 2012); secondly, decomposing these interactions into “important components” (Lee and Tzou, 2009); and thirdly, carrying out cellular-systems modeling and analysis (Nobu et al., 2015; Mcgeachie et al., 2016). It is easy to see how such an approach could also be integrated with the mathematical models of the rumen environment described earlier.

Network-based approaches are likely to provide the best hope of integrating all this information by allowing a complete overview of the rumen microbiome as they are capable of capturing the fundamental properties of the microbial ecosystem including taxonomy (Barberan et al., 2012), functional similarity (Martiny et al., 2015), metabolic processes (Shlomi et al., 2008), co-expression (Zhang and Horvath, 2005) and gene-sharing (Smillie et al., 2011). Network-based approaches have proven successful for generating insight into the functional and genetic potential of microbial communities, bringing clarity to the complex history of microbes, providing tools that allow analyses of mosaic sequences (Adai et al., 2004), and identify genomes harboring sequences of multiple origins (Lima-Mendez et al., 2008; Fondi and Fani, 2010; Kloesges et al., 2011; Halary et al., 2013). Network-based approaches also provide a framework where genetic diversity can be compared and quantified, even when analyzing highly divergent sequences (Bhattacharya et al., 2013). We are only in the early stages of applying these approaches to the rumen microbiome, and while network scaffolds for specific animal studies have been generated (Roehe et al., 2016), large-scale, integrative models have yet to be developed.

## Conclusions

The terminology of microbiome is often misused within the rumen context as there is a tendency to focus on the study of the rumen bacteria alone, without consideration of the eukaryotes. This undoubtedly limits our ability to understand the rumen microbiome, as these groups of microbes interact with each other, consequently affecting production and the environmental impact of ruminants (Table 4). Also, little emphasis is given to understanding ecological interactions, niche specialization and consequences of the biofilm phenotype and membrane vesicles on animal phenotype. An increased fundamental understanding of the rumen microbiome as well as that of the lower GI tract of ruminants is essential to develop novel approaches for improving livestock production and reducing environmental impact. Irrespective, much emphasis has been given to using plant/additive/supplement strategies to manipulate the rumen microbiome in a manner that improves efficiency of animal production. Although, these have had limited success when applied to the mature animal, recent data show that ruminant breeding programs coupled with a defined dietary management protocols from birth may be the best ways to achieve lifelong animal phenotype benefits with limited financial and labor input. Further research is required to assess the role of the host genome on the rumen microbiome and animal phenotype on a global geographic scale (Table 4). Likewise, application of these innovative techniques to early life ruminant nutrition is in its infancy, and a global effort is required to define the best practices in early life which will differ based on geography (Table 4). Improving our ability to measure phenotype using rumen microbiome gene based biomarkers may also allow high throughput phenotype predictions (Table 4). Irrespective, the ruminant sector has made major strides to improving animal phenotype, through understanding the rumen microbiome, and further advances will no doubt be accomplished, especially given the exponential advances in “omic” technologies.

**Table 4:** Outstanding questions.

What role do the rumen eukaryotome play in animal phenotype? What role do phages play in shaping the rumen microbiome? How can we harvest the ability of the rumen microbes to produce biofilms and membrane vesicles to address global livestock challenges? Are early life dietary interventions effective in enhancing animal phenotype in the long-term? What role do the lower GI tract microbiomes potentially play in the animal phenotype? Can we use host genomics and develop ruminant breeding programs to beneficially manipulate the rumen microbiome to enhance animal phenotype? How effective would a strategy involving enhanced ruminant genomics coupled with effective early life dietary management of the animal be in enhancing animal phenotype? Is mathematical modeling a feasible accurate approach to predicting ruminant feed efficiency and methane output? Can we develop gene based biomarkers to predict feed efficiency and/or methane output from ruminants? How resilient are ruminants with an improved phenotype to perturbations e.g., acidosis? Given that preliminary data shows that these animals have a less diverse rumen microbiome? How can we ensure comparability of data generated globally to form a consensus on best practices for achieving the global livestock challenges?

## Author contributions

All authors listed have made a substantial, direct, and intellectual contribution to the work and approved it for publication.

### Conflict of interest statement

The authors declare that the research was conducted in the absence of any commercial or financial relationships that could be construed as a potential conflict of interest.
